# Type II enteropathy-associated T-cell lymphoma features a unique genomic profile with highly recurrent *SETD2* alterations

**DOI:** 10.1038/ncomms12602

**Published:** 2016-09-07

**Authors:** Annalisa Roberti, Maria Pamela Dobay, Bettina Bisig, David Vallois, Cloé Boéchat, Evripidis Lanitis, Brigitte Bouchindhomme, Marie- Cécile Parrens, Céline Bossard, Leticia Quintanilla-Martinez, Edoardo Missiaglia, Philippe Gaulard, Laurence de Leval

**Affiliations:** 1 University Institute of Pathology, Service of Clinical Pathology, Centre Hospitalier Universitaire Vaudois, 25 rue du Bugnon, 1011 Lausanne, Switzerland; 2 SIB Swiss Institute of Bioinformatics – Quartier Sorge, bâtiment Génopode, 1015 Lausanne, Switzerland; 3 Ludwig Cancer Research Center Lausanne, Chemin des Boveresses 155, Biopôle III, 1066 Epalinges, Switzerland; 4 Institute of Pathology, CHR-U de Lille/Université de Lille II, Avenue Oscar Lambret, 59037 Lille Cedex, France; 5 Department of Pathology, CHU de Bordeaux, Hopital du Haut Lévêque, Avenue Magellan, 33604 Pessac, France; 6 Department of Pathology, CHU de Nantes – Hôtel Dieu, 9 quai Moncousu – Plateau technique 1, 44093 Nantes, France; 7 Institute of Pathology and Neuropathology, Eberhard Karls University of Tübingen and Comprehensive Cancer Center, University Hospital Tübingen, Tübingen 72076, Germany; 8 Department of Pathology, Hôpital Henri Mondor, AP-HP, INSERM U955, and University Paris-Est, 51 Avenue du Mal de Lattre de Tassigny, 94010 Créteil, France

## Abstract

Enteropathy-associated T-cell lymphoma (EATL), a rare and aggressive intestinal malignancy of intraepithelial T lymphocytes, comprises two disease variants (EATL-I and EATL-II) differing in clinical characteristics and pathological features. Here we report findings derived from whole-exome sequencing of 15 EATL-II tumour-normal tissue pairs. The tumour suppressor gene *SETD2* encoding a non-redundant H3K36-specific trimethyltransferase is altered in 14/15 cases (93%), mainly by loss-of-function mutations and/or loss of the corresponding locus (3p21.31). These alterations consistently correlate with defective H3K36 trimethylation. The JAK/STAT pathway comprises recurrent *STAT5B* (60%), *JAK3* (46%) and *SH2B3* (20%) mutations, including a STAT5B V712E activating variant. In addition, frequent mutations in *TP53*, *BRAF* and *KRAS* are observed. Conversely, in EATL-I, no *SETD2*, *STAT5B* or *JAK3* mutations are found, and H3K36 trimethylation is preserved. This study describes *SETD2* inactivation as EATL-II molecular hallmark, supports EATL-I and -II being two distinct entities, and defines potential new targets for therapeutic intervention.

## Abstract (web summary)

Enteropathy associated T-cell lymphoma -EATL- affects the intestine and there are two different subtypes. In this study, the authors carry out exome sequencing of the type II variant and find that it is characterized by recurrent mutations in the histone methyltransferase SETD2 that are accompanied by altered H3K6 methylation.

## Graphical abstract

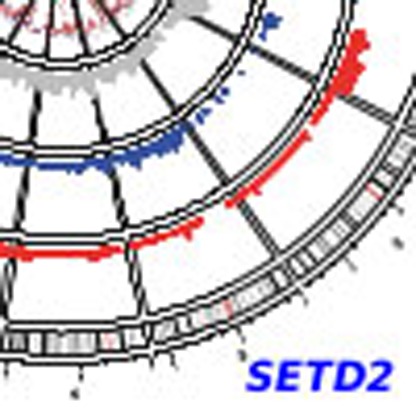

Enteropathy-associated T-cell lymphoma (EATL) is a rare peripheral T-cell lymphoma entity derived from intraepithelial intestinal cytotoxic T lymphocytes^1^. The current WHO classification recognizes two disease variants, which differ by epidemiology, clinical features, morphology and immunophenotype: type I (EATL-I), and type II (EATL-II). EATL-I, most frequent in Europe and often associated with coeliac sprue, usually features pleomorphic morphology comprising medium to large cells with frequent CD30 expression, an inflammatory component, and frequent necrosis. EATL-II, is less common overall, but in contrast to EATL-I infrequently associates with coeliac sprue and occurs worldwide. EATL-II also termed monomorphic CD56+ intestinal T-cell lymphoma, or monomorphic epitheliotropic intestinal T-cell lymphoma^2^, is composed of CD3+ CD8+ CD56+ monomorphic medium-sized cells with round nuclei and pale cytoplasm, showing marked epitheliotropism to the adjacent intestinal mucosa. In the majority of cases the neoplastic cells are derived from γδ-T cells, although some cases are of αβ-T-cell derivation and a minority of cases may be either double positive or double negative for T-cell receptor (TCR) beta and TCR gamma expression^1^,^3^,^4^,^5^. An inflammatory infiltrate is typically absent and necrosis is less frequent than in EATL-I. Both subtypes are highly aggressive disorders with 5-year survival rates below 20% (ref. 6) and few treatment options available.

The genetic basis of EATL-II still remains poorly characterized, mainly restricted to copy number alterations^7^,^8^,^9^ and targeted sequencing studies^10^,^11^. Comparative genomic hybridization (CGH)-based studies have shown that both types share common recurrent chromosomal imbalances, and also distinctive genetic alterations. Regardless of the subtype, EATL is cytogenetically characterized by chromosome 9q gains and almost mutually exclusive losses of 16q12.1. Gain of chromosome 7 and losses of 8p22-23.2, 16q21.1, 11q14.1-q14.2 and 9p21.2-p21.3 are also frequent in both EATL variants. Conversely, EATL-II is characterized by significantly more frequent gains of the MYC oncogene locus and less-frequent gains of chromosomes 1q and 5q as compared with EATL-I, suggesting two distinct genetic pathways. In addition, the loss of 3p21.31 has been reported as recurrent in type II but not in type I EATL.

Recent works have pointed at the implication of the JAK/STAT pathway in EATL-II. Recurrent mutations of *STAT5B* in EATL-II were first reported in 2015 and found to occur exclusively in cases of γδ origin^11^. The study by Nairismagi *et al*.^10^ further substantiated recurrent mutations mainly affecting hotspots in *STAT5B* and *JAK3*.

Here we sought to define the genetic landscape of EATL-II by performing whole-exome sequencing (WES) on a series of 15 paired tumour-normal EATL-II samples. The highlight of our study is the discovery of frequent alterations of the tumour suppressor *SETD2* gene by loss-of-function mutations and/or loss of the corresponding 3p.21 locus in the majority of the cases (14/15). Our findings also further expand the documentation of frequent improper activation of the JAK/STAT5 pathway, principally by recurrent mutations in *STAT5B* (60%), *JAK3* (46%) and *SH2B3* (20%). In addition, we show that in EATL-I, SETD2 alterations are not found while a distinct pattern of JAK/STAT mutations is observed, supporting that both EATL variants correspond to distinct pathological entities.

## Results

### Genomic landscape of EATL-II

The outline of our study is depicted in Supplementary Fig. 1. For the majority of our cases only formalin-fixed, paraffin-embedded (FFPE) tissue was available for analysis. Thus, starting from the notion that fresh-frozen material represents a benchmark for WES technique, as a first step we validated the quality of our WES protocol and bioinformatics pipeline on FFPE samples by comparing two paired normal/tumour DNA samples of intestinal lymphomas for which both fresh-frozen and FFPE tissue samples were available (one EATL-II, corresponding to case 1 of our cohort, and one intestinal ALK-positive anaplastic large cell lymphoma; Methods and Supplementary Figs 2 and 3). Having shown a very good concordance between results generated from fresh-frozen and FFPE materials, we then performed WES of 15 paired tumour-normal DNA samples isolated by microdissection of routinely processed FFPE EATL-II surgical samples (Supplementary Table 1).

**Figure 1:**
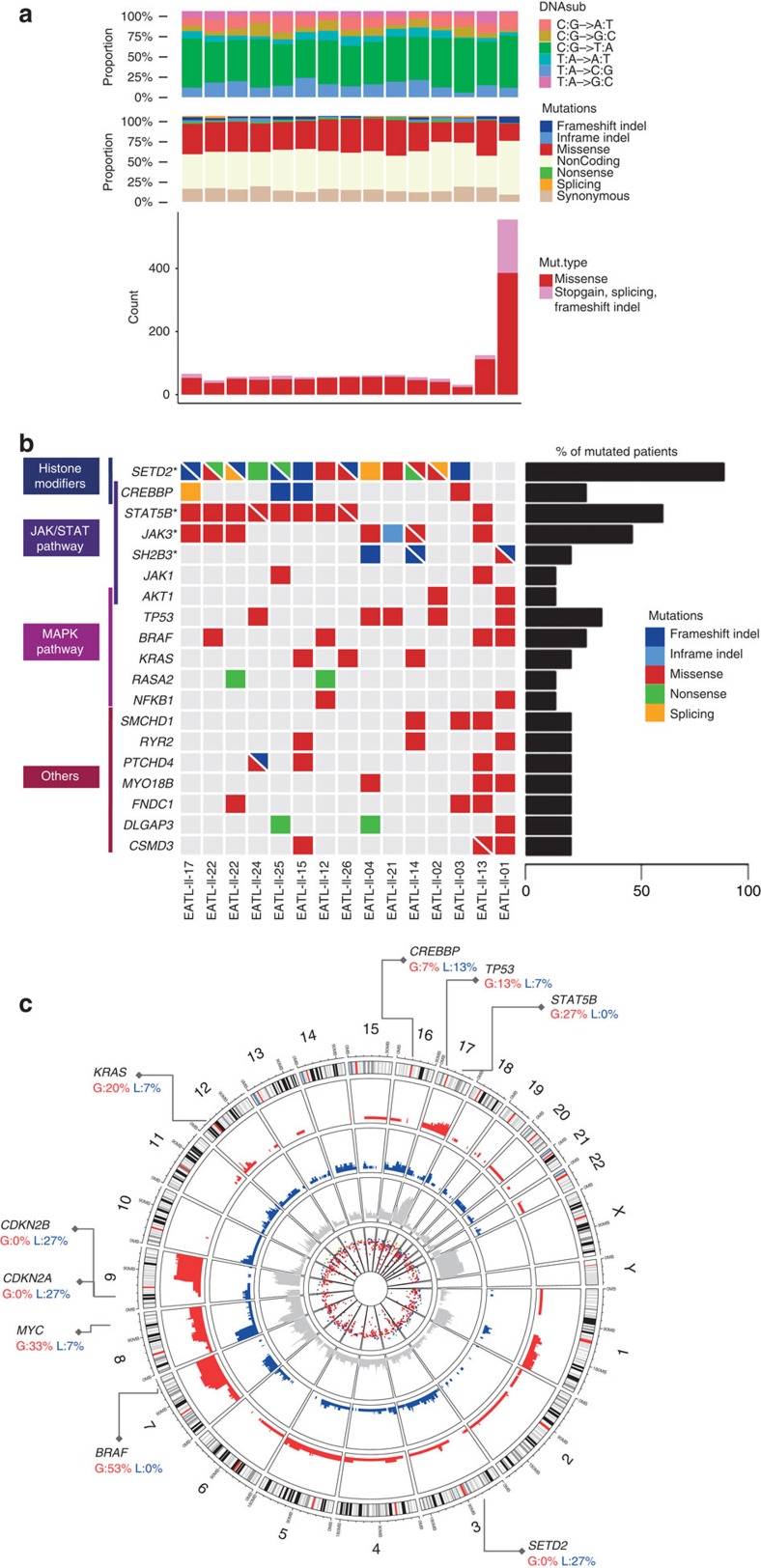
Mutational landscape of EATL-II identified by whole-exome sequencing. (**a**) The proportion of transitions and transversions, the proportion of each type of mutations, and the number of non-silent mutations, are shown individually for each of the 15 EATL-II samples analysed. The median number of non-synonymous mutations was 57 per patient. Sample EATL-II-01, showing the highest number of mutations, originated from a recurrent tumour in a patient previously treated with chemotherapy. Overall, missense mutations represented the most frequent type of mutation. (**b**) Mutations in genes mutated in more than 3/15 patients (≥20%) and in a selection of frequently mutated genes among statistically significantly enriched pathways (≥2 patients) are represented for each of the 15 EATL-II samples analysed and arranged by functional groups. Samples are displayed as columns and mutations are coloured by the type of alteration. Double mutations are represented as triangles, single mutations as full coloured squares. The percentage of mutated samples is represented on the right. Genes found significantly recurrently mutated by MutSigCV algorithm with a false discovery rate (FDR) below 15% are marked with an asterisk (*). (**c**) The Circos diagram summarizes the somatically acquired genetic variants in EATL-II genome detected by WES. From outermost to innermost tracks: (1) ideogram representing chromosomes oriented clockwise, with centromeres indicated in red; (2–3) plot of somatic copy number alterations and relative frequency: gains (red) and losses (blue); (4) neutral LOH distribution; (5) distribution of somatic mutations and different mutation types across the genome colored by type of alteration. A selection of significant genes is reported.

WES produced a mean coverage of 195 × and 213 × for tumour and normal samples, respectively, with around 89% of the target sequence covered by at least 50 reads. Overall, we found a total of 3,488 somatic mutations, including 1,393 non-silent mutations in 1,205 genes, with a median of 57 (range 31–555) somatic non-synonymous mutations per patient (average of 1.7 per Mb). As in other tumours, C>T transitions were the most frequent exonic mutations (∼50%), followed by C>A transversions (12%) (Fig. 1a and Supplementary Data 1). Validation performed by Sanger sequencing and/or targeted deep sequencing on 59 variants selected from the most relevant genes, showed 91% concordance across the different methods used (Supplementary Data 1 and Supplementary Tables 2 and 3).

The mutational landscape of EATL-II was remarkably homogeneous showing significantly enriched clusters among histone modifier genes, JAK-STAT and MAPK-signalling pathways (Fig. 1b and Supplementary Data 2). The Histone-lysine *N*-methyltransferase *SETD2* gene on chromosome 3p21.31 was mutated in 13/15 (86%) tumours. We also observed predicted deleterious mutations in *CREBBP* histone acetyltransferase in 26% of the cases and we found a broad spectrum of mutations in other epigenetic genes previously described as frequently mutated in B-cell lymphomas like *EP300, EZH2 and ARID1* (refs 12, 13, 14). Conversely no recurrent mutations in epigenetic regulatory genes, like *TET2, DNMT3A* and *IDH1/2* were observed, while these genes are commonly mutated in other peripheral T-cell lymphoma entities^15^,^16^,^17^. All the patients analysed harboured at least one mutation in the JAK/STAT axis with recurrent mutations in *STAT5B* (60%), *JAK3* (46%) and in the negative regulator of JAK signalling *SH2B3* (20%). We furthermore observed frequent alterations in the MAPK pathway in 80% of EATL-II cases, including *TP53* (33%), *BRAF* (26%), *KRA*S (20%) and *NRAS* mutations which were essentially mutually exclusive.

Copy number analysis derived from WES data identified multiple regions of frequent gains and losses, essentially consistent with previous array CGH studies^8^,^9^. In particular, we observed gains in 9q (70% of the cases), 7q (60% of the cases), and 8q24 (comprising the *MYC* locus, in 35% of the cases), and recurrent losses involving 8p, 16q, 11q, and 9p21.3 (corresponding to the genomic localization of *CDK2A/B* tumour suppressor genes, lost in 20% of the cases) (Fig. 1c, Supplementary Fig. 4 and Supplementary Data 3). Interestingly some of the recurrently mutated genes mapped to chromosomal regions of gains (*BRAF* locus at 7q34) or losses (*SETD2* locus at 3p21.31).

### Highly recurrent *SETD2* alterations in EATL-II

**Figure 2:**
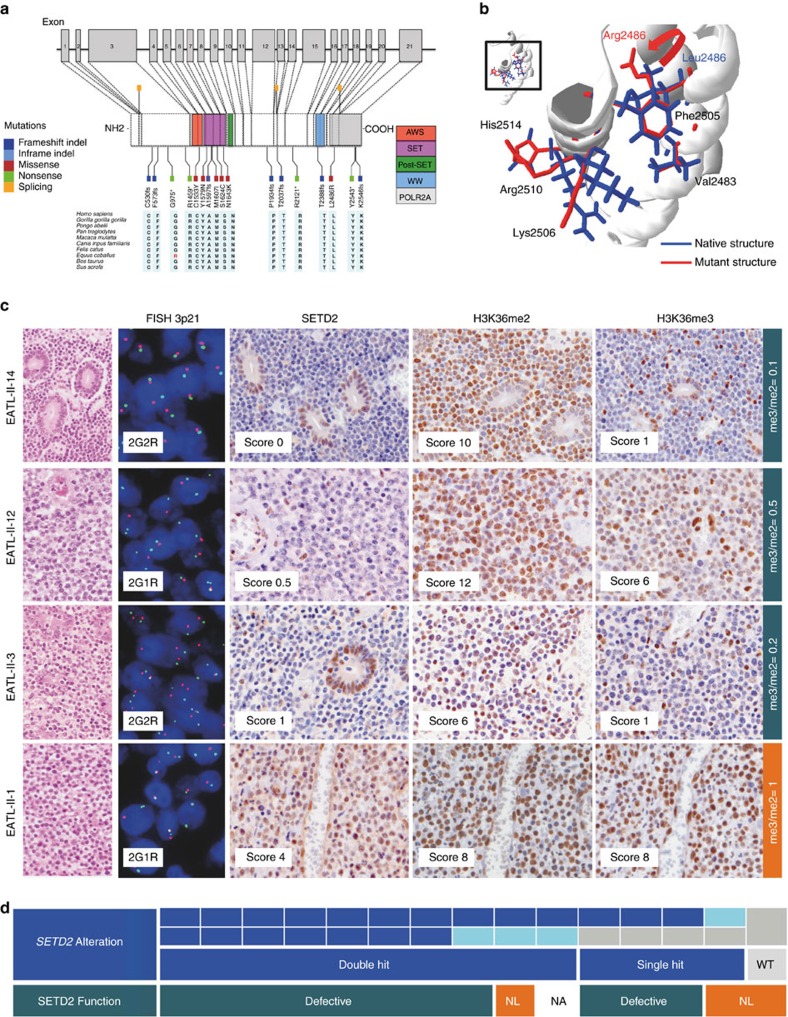
Recurrent *SETD2* alterations in EATL-II. (**a**) *SETD2* mutations observed in EATL-II samples analysed by WES. The locations of the AWS, SET, post-SET and WW domains of SETD2 are indicated. The region of interaction with RNA pol-II is also represented. The type and position of each identified mutation is shown. Across species, homology of SETD2 amino-acid changes was determined by multiple sequence alignment. The high level of conservation of these sites among species indicates that the *SETD2* mutations all target conserved residues. (**b**) Computational modelling of the L2486R SET2 variant. The L2486R residue lies within one of the critical alpha helices structurally supporting the RNA pol-II binding site. This mutation was consistently predicted to be destabilizing (DG>0 kcal mol^−1^) with structure-based methodologies (predicted DG between 2.35 to >10 kcal mol^−1^). Note the predicted changes in side chains of critical SRI domain residues. (**c**) Morphology (haematoxylin and eosin, original magnification × 400), FISH (3p21.31 locus, original magnification × 630) and immunohistochemical stainings for SETD2, H3K36me2 and H3K36me3 (immunoperoxidase, original magnification × 400) in four EATL-II cases with different types of *SETD2* gene alterations (EATL-II-14: double hits missense and nonsense mutations; EATL-II-12: missense mutation and heterozygous 3p21.31 deletion; EATL-II-3: one frameshift indel mutation; EATL-II-1: no mutations but heterozygous 3p21.31 loss). R and G denote ‘red' and ‘green' signals to indicate the most common FISH hybridization pattern seen in each nucleus. (**d**) Summary of *SET2* gene alterations and SET2 function in the 15 EATL-II analysed by WES. Mutations of *SET2* are represented in dark blue and *SET2* locus deletion in light blue. NA, not analysed; NL, not lost.

*SETD2* was found to be the most significantly recurrently mutated gene (MutSigCV *P* value=2.5 × 10^−15^ and a false discovery rate=4.8 × 10^−11^), observed in 86% (13/15) of EATL-II tumours with 20 distinctive mutations. Fourteen of these mutations consisted of premature stop codon, non sense, frameshift indels or splicing mutations expected to confer critical changes in protein structure. The other six missense mutations occurred in highly conserved residues of functional domains, and were predicted to be deleterious with a damaging effect on the protein by SIFT and Polyphen2 algorithms (Fig. 2a and Table 1).

We performed three-dimensional (3D) modelling and structure stability analyses for all missense mutation covered by the only available SET and AWS (PDB-ID 4FMU) and SRI (PDB-ID 2A7O) domain-containing templates (Fig. 2b and Supplementary Fig. 5). The L2486R alteration on sample 21, the only case with a monoallelic missense mutation, was consistently predicted as the most destabilizing mutation (predicted ΔG range from 2.35 to >10 kcal mol^−1^, Fig. 2b). It occurs on a critical alpha helix on the RNA pol-II binding domain, and SETD2 binding to RNA pol II can be impaired by the destabilized structure (Fig. 2b, Supplementary Fig. 5c)^18^. All other mutations indicated destabilizing effects except S1624C under SDM^19^ and Eris^20^; S1624C is nonetheless predicted as disease causing in SDM. All mutations, except the Y1579 mutation (case 22), have been functionally confirmed by IHC to lead to impaired H3K36 trimethylation. The Y1579N mutation occurs in a highly conserved region of the SET binding pocket, and ranks third in terms of predicted effects on stability; nonetheless, no reduction in H3K36 trimethylation was found in the mutated case, indicating that the phenotype that we see cannot be explained as a function of structural alteration.

Interestingly, among the 13 *SETD2*-mutated cases, seven harboured double mutations in *SETD2*. Moreover, CNV analysis showed heterozygous deletion of the *SETD2* locus (3p21.31) in four cases, of which three had a concurrent monoallelic mutation in *SETD2* and one had no detectable *SETD2* mutation. To validate the CNV analysis, we developed original FISH probes designed to identify deletions of the *SETD2* locus at 3p21.31 by selecting two BAC clones hybridizing to the *SETD2* locus and two BAC clones hybridizing to a preserved region on chromosome 3 (see Methods and Supplementary Fig. 6). FISH analysis confirmed heterozygous deletion of 3p21.31 locus in the four cases as predicted by CNV analysis and no deletion in the other 11 cases (Fig. 2c, and Table 1). Overall 10/13 (76%) *SETD2*-mutated patients seemed to have double genetic hits affecting the gene (Fig. 2d). However, the biallelic inactivation of *SETD2* could only be confirmed in the three cases with heterozygous deletion of *SETD2* locus and concurrent mutation in the remaining allele. In the other seven cases with double mutations, the paired mutations were too far apart to be visualized by either sequencing chromatograms or BAM files, and hence their biallelic nature could only be speculated.

### Defective H3K36 trimethylation in EATL-II

*SETD2* encodes a methyltransferase that in humans is non-redundantly responsible for specifically trimethylating ‘Lys-36' of histone H3 (H3K36me3) using dimethylated ‘Lys-36' (H3K36me2) as a substrate^21^. To determine the functional consequences of *SETD2* alterations, we performed immunohistochemistry (IHC) for semi-quantitative assessment of the SETD2–H3K36me2–H3K36me3 axis at the protein level (Fig. 2c,d and Table 1). Briefly, both the extent of immunostainings (in quartiles from 0 to 100%, scores 0 to 4) and staining intensity (scores 0 to 3) were evaluated and multiplied to provide an IHC score on a scale ranging from 0 to 12. As a control, we also performed IHC for total H3 histone, which consistently showed strong homogeneous staining in most cells (score 12) in 10/10 cases tested.

Among the 13 *SETD2*-mutated cases, SETD2 IHC showed either absent or very low expression levels of the protein (score 0 to 3) in 9/10 evaluable cases, and a score 6 in one case (median score=0.75). H3K36me2 showed consistent nuclear staining in 12/12 evaluable *SETD2*-mutated cases, with IHC scores ranging from 4.5 to 12 (median score=8). In *SETD2*-mutated tumours H3K36me3 had IHC scores ranging from 0 to 12 (median score 1) being either undetectable or very weakly expressed (scores 0 to 2) in 10/12 evaluable cases. Given the variation in H3K36me2 IHC scores, we also calculated in each case the ratio between H3K36me3 and H3K36me2 IHC scores, which ranged from 0 to 1.5 (median 0.1) in *SETD2*-mutated cases. In summary, the pattern of immunostaining in most of the SETD2-mutated cases was low SETD2 expression, moderate to strong H3K36me2 expression, and defective H3K36 trimethylation indicated by absent or weak detection of H3K36me3. Accordingly, the H3K36me3/me2 ratio was <1 in most cases. One sample (case 22) with double mutations in *SETD2* (a nonsense truncating mutation co-occurring with a missense mutation) had an unexpected profile. In accordance with the genotype, this case was characterized by low SETD2 expression; however, the preserved trimethylation observed for H3K36 (IHC score 12) was discordant with both the genotype and the reduced level of SETD2 (IHC score 2) (Supplementary Fig. 7). Notably, the fact that three tumours with a monoallelic *SETD2* mutation showed a strong reduction in H3K36me3 level suggests molecular alterations with a dominant phenotype.

Recent data demonstrated that total H3K36me3 levels are not significantly impacted by monoallelic loss of *SETD2* (ref. 22). Accordingly in the case with a single copy loss of the *SETD2* locus, and wild type remaining allele, SETD2 protein expression was reduced (IHC score 4) but its function appeared to be preserved (case 1) (Fig. 2c). In the only *SETD2*-wild type case with no deletion of the genomic locus (case 13) SETD2 was diffusely expressed and both H3K36me2 and H3K36me3 featured high IHC scores (Supplementary Fig. 7).

Overall, these data strongly support a direct correlation between *SETD2* mutations and protein loss of function in EATL-II, and indicate that reduced H3K36me3 levels may represent an important step in EATL-II lymphomagenesis.

### JAK/STAT pathway activation in EATL-II

We found that all 15 EATL-II cases examined by WES showed mutations in one or more genes of the JAK/STAT pathway, with frequent coexisting CNV or loss of heterozygosity (LOH) of the corresponding genomic loci (Supplementary Fig. 8).

*STAT5B* mutations were identified in nine samples (60%) (single mutations in seven cases and double mutations involving the same allele in two cases) and almost exclusively associated with LOH preserving the mutated allele, suggesting that the variants predominate in the tumour tissue. Specifically, LOH of *STAT5B* locus, involving all or part of chromosome 17, was observed in 10 out 15 EATL-II cases analysed by WGS (Supplementary Fig. 8a), 6 of which are copy-neutral. To support our findings, LOH status was confirmed by TaqMan SNP genotyping assay targeting a polymorphic site (rs1126821) 439 kb upstream to the *STAT5B* genomic locus in five cases (Supplementary Fig. 9).

**Figure 3:**
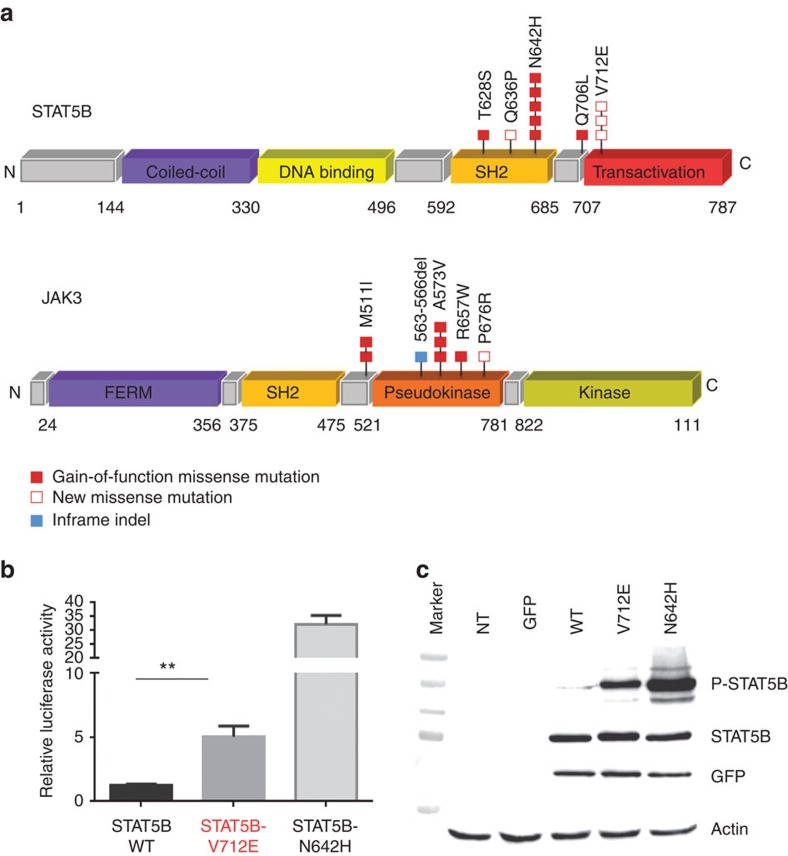
Recurrent alterations targeting the JAK/STAT pathway in EATL-II. (**a**) Schematic representation of somatic alterations in *STAT5B* and *JAK3* identified in this study. The functional domains of the proteins are shown. Filled rectangles represent mutations that have been previously described (red: missense mutation and blue: indel). Empty rectangles indicate previously unreported mutations. (**b**) Luciferase assay was performed in Hela cells co-transfected with the STAT5B constructs (WT, V712E and N642H) or empty vector together with a luciferase sequence under the control of a STAT5-binding element. Luciferase assay was performed in three independent experiments. Each condition was tested in triplicate and the fold change was normalized with the relative luciferase activity of the empty vector. Each column represents mean±s.d. of three independent experiments. Both mutants clearly induced increased luciferase activity when compared with the wild-type STAT5B plasmid. (**c**) P-STAT5(Y699) and STAT5 protein expression levels in Hela cells transfected with STAT5B wild-type or mutant constructs V712E and N642H are shown by western blotting. Non-transfected cell line (NT) and cell line transfected with an empty vector (GFP) were used as controls for the reaction. STAT5B variants (V712E and N642H) led to increased phosphorylation of STAT5B as compared with the ectopic expression of the STAT5B-WT. The transduction efficiency for each condition was assessed by total STAT5B and GFP expression. One experiment representative of three independent experiments is shown. Western blotting results were congruent with the luciferase assay, confirming the constitutive hyperphosphorylation of both N642H and V712E STAT5B variants.

In addition to the gain-of-function STAT5B N642H variant previously reported in EATL-II of γδ derivation^11^, we found other *STAT5B* hotspot activating mutations and a novel STAT5B V712E variant in three tumours (Supplementary Data 1 and Fig. 3a). Accordingly, we sought to investigate the functional effect of the p.V712E on JAK-STAT5 activation. In HeLa cells transfected with STAT5B-mutant constructs, the expression of both N642H and V712E variants led to increased STAT5B transcriptional activity and to constitutive STAT5B hyperphosphorylation compared with the wild type (Fig. 3b), validating the V712E variant as a new somatic gain-of-function mutation. Six of the nine *STAT5B*-mutated cases were characterized with certainty as for TCR isoform expression and comprised three γδ TCR-positive tumours, two αβ TCR-positive tumours and one case positive for both TCR isoforms (Supplementary Fig. 8a).

*JAK3* mutations found in 7/15 (46%) EATL-II cases included several activating variants mainly in the pseudokinase domain (A573V, M511I, R657W, K563-566del) (Fig. 3a)^14^,^23^,^24^,^25^ and a novel P676R alteration which co-occurred with the M511I variant in one case. Three cases (20%) harboured loss-of-function mutations in *SH2B3*, a negative regulator of the JAK–STAT pathway^26^. Two cases harboured distinct *JAK1* mutations. Alterations in other components of the pathway were not recurrent.

### Targeted analysis of additional intestinal lymphomas

Next, we wanted to validate our WES discoveries by extending our analyses to additional EATL-II cases, and to investigate if the characteristic genetic aberrations of SETD2, STAT5B and other JAK/STAT elements found in EATL-II were shared by EATL-I. For that purpose we collected an extended cohort composed of 14 additional EATL-II, 11 EATL-I and 8 non-EATL intestinal lymphomas of various histologies. For targeted sequencing, we designed amplicon pools (Supplementary Table 3) specifically interrogating the top genes mutated in EATL-II and a selection of hotspot mutations in the JAK/STAT pathway known to be recurrent mutated in other hematopoietic and lymphoid tumours.

**Figure 4:**
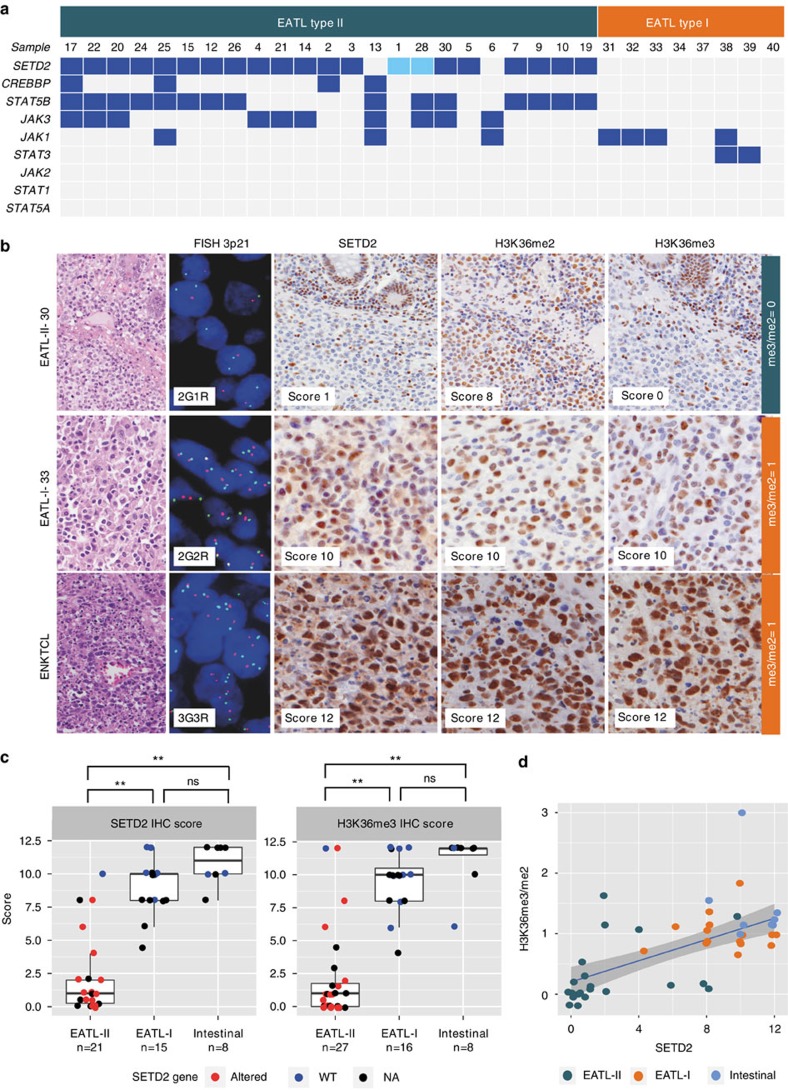
EATL-II has a genetic profile distinct from EATL-I and other intestinal lymphomas. (**a**) Summary of mutations in a selected panel of genes examined by targeted deep sequencing in 23 EATL-II and 8 EATL-I cases. Mutations are represented in dark blue. Loss of *SETD2* locus is reported in light blue when present in the absence of mutation (**b**) Morphology (haematoxylin and eosin, original magnification × 400), FISH (3p21.31 locus, original magnification × 630) and immunohistochemical stainings for SETD2, H3K36me2 and H3K36me3 (immunoperoxidase, original magnification × 400) in three intestinal lymphomas representative of EATL-II, EATL-I and other histotypes (ENKTCL: extranodal NK/T-cell lymphoma). R and G denote ‘red' and ‘green' to indicate the most common FISH hybridization pattern seen in each nucleus. (**c**) Immunohistochemistry results for SETD2 and H3K36me3 levels in EATL-II, EATL-I and other intestinal lymphomas. The *SETD2* gene status is colour-coded. Red indicates *SETD2* alteration (heterozygous loss and/or mutation(s)); blue, *SETD2*-WT; black, unknown *SETD2* mutation status. Wilcoxon signed-rank test, ^**^highly significant (*P* value <0.001), NS, non significant (*P* value >0.05). The top, the middle and the bottom lines of the boxes show the first, the second (median) and the third quartiles of the proteins score, respectively. Whiskers represent the scores that are 1.5 times the interquarile range (IQR) above or below the first and the third quartiles. (**d**) H3K36me3 levels normalized to those of H3K36me2 (H3K36me3/me2 ratio) were plotted against SETD2 IHC score for all patients of the extended cohort with complete characterization (21 EATL-II, 15 EATL-I and 8 other intestinal lymphomas). A highly significant correlation was observed between SETD2 expression and H3K36me3/me2 ratio score (Spearman's rank correlation; *P* value <0.001).

Targeted deep sequencing was applied to the 15 EATL-II cases of the discovery cohort and essentially confirmed the mutations identified in the top mutated genes (Supplementary Table 4). It could also be performed on 8/14 additional EATL-II tumours and revealed *SETD2* mutations in 6/8 cases, STAT5B mutations in 6/8 cases, and JAK3 and JAK1 mutations in three and one case, respectively (Supplementary Table 5 and Fig. 4a). FISH analysis of the 3p.21 locus was evaluable in 7/8 cases and showed heterozygous deletion of the locus in three cases, of which two had a *SETD2* mutation and one was *SETD2*-wt. Consistent with previous observations on the WES cohort, all six *SETD2*-mutated cases showed overall low levels of expression of SETD2 and H3K36me3 (Fig. 4b and Supplementary Table 6). All *STAT5B* mutations were observed in cases harbouring *SETD2* alterations. Taking into account the cases of the WES cohort and the additional eight cases with targeted sequencing, interestingly, the two tumours with no alterations in SETD2 (case 13 and case 6) both harboured concurrent mutations in *JAK1* and *JAK3*.

Interestingly, no *SETD2*, *STAT5B*, *JAK3* or *CREBBP* mutations were identified in any of the eight EATL-I cases analysed by targeted deep sequencing (Fig. 4a and Supplementary Table 5). Furthermore, in four out of eight EATL-I tumours, *JAK1* was affected by missense mutations at the same amino-acid residue G1097, a position that has been reported as frequently mutated in anaplastic large cell lymphomas^27^, and two harboured hotspot gain-of-function *STAT3* mutations (N647I and K658N)^28^.

Overall, considering the entire cohort of cases included in this study (15 WES EATL-II plus the extended cohort), FISH analysis of the *SETD2* locus revealed 3p21.31 loss in 7/24 contributive EATL-II cases, in 0/11 evaluable EATL-I cases and in 0/8 intestinal lymphomas of other histotypes (Supplementary Table 6 and Fig. 4b). Moreover, IHC showed that both SETD2 and H3K36me3 scores were significantly higher in EATL-I and other intestinal lymphomas, compared with EATL-II (Wilcoxon signed-rank test, *P* value <0.001) (Fig. 4b,c). SETD2 expression was highly correlated to H3K36me3/me2 ratio (Spearman's rank correlation, Rho=0.64, *P* value <0.001) (Fig. 4d), with most EATL-II clustering at the lower end of the regression line. In contrast, EATL-I and other intestinal lymphomas exhibited high levels of SETD2 protein expression and a H3K36me3/me2 ratio close to 1, indicating preserved SETD2 catalytic activity.

## Discussion

Our study comprising the largest EATL-II cohort analysed by WES, provides a comprehensive genomic characterization of this rare and aggressive lymphoma. The highlight of our findings is the discovery of highly recurrent alterations of the *SETD2* gene by mutations and/or loss of the corresponding 3p.21 locus in >90% of the cases (21/23 cases examined by WES or targeted deep sequencing), bringing to the light a previously unexplored genetic player in the pathogenesis of this disease.

*SETD2* encodes a lysine methyltransferase responsible for histone H3 lysine 36 trimethylation (H3K36me3), which interacts with RNA polymerase II and is recruited to sites of active transcription^29^. Among various genes coding for enzymes with methytransferase activity, *SETD2* shows a very high frequency of somatic mutations in various cancer types, with high incidence of deleterious and loss-of-function mutations supporting its tumour suppressor function^29^. The tumour suppressor role of SETD2 is emerging in several cancer types, including clear cell renal cell carcinoma, breast and lung cancers, acute lymphoblastic leukaemia, high-grade gliomas and gastrointestinal stromal tumours^30^,^31^,^32^,^33^,^34^. SETD2 inactivation has been demonstrated as a critical event in facilitating both disease initiation and progression through decreasing H3K36me3 (ref. 33).

Our study defines the genetic inactivation of *SETD2* as a hallmark of EATL-II, and shows for the first time highly prevalent *SETD2* deregulation in a cancer of mature lymphocytes. Of 23 EATL-II cases with complete genomic *SETD2* assessment, 19/23 (83%) were *SETD2*-mutated, 7/23 (30%) had loss of one *SETD2* locus, and overall 21/23 (91%) had *SETD2* genomic alteration. The high frequency of *SETD2* alterations and the mutational spectrum strongly supports the tumour suppressor role of SETD2 in EATL-II. In fact, we found that 18 of the total 26 mutations (69%) identified in our *ETAL*-II cases were either nonsense (6) frameshift indels (9) or splicing mutations (3) predicted to impair protein function and distributed along the whole gene. Instead, all the missense mutations tended to cluster in functional domains of the SETD2 protein and were predicted to be probably damaging. Although only five mutations have been previously described in COSMIC (Supplementary Data 1 and Supplementary Table 5), the *SETD2* mutational spectrum in EATL-II well overlapped with the pattern of mutations found in other cancer types^33^,^34^,^35^. In accordance with the notion that multiple hits on the same gene are generally required for inactivating the function of tumour suppressor gene, we found that the majority of the cases (12/21) had double genetic hits affecting *SETD2* either by double mutations (7/12) or concurrent 3p21.31 loss and *SETD2* mutation in the remaining allele (5/12). Although for technical reason we could not formally demonstrate that the double SETD2 mutations corresponded to mutations on both alleles, we can reasonably speculate that these mutations are biallelic. In fact biallelic inactivation of *SETD2* gene has been previously demonstrated in other cancer types by both double mutations occurring on separate alleles of SETD2 in individual patients with acute leukaemias^33^ or by concurrent 3p loss and loss-of-function SETD2 mutation in the remaining allele in renal cell carcinomas^22^. We additionally showed that *SETD2* mutations overall correlate with complete loss or strong reduction in H3K36me3, suggesting that these are indeed loss of function. Interestingly, we observed that not only double inactivating hits but also monoallelic *SETD2* mutations impaired histone H3K36 trimethylation supporting the notion of a dominant phenotype. This observation is in accordance with findings reported by other groups in high-grade gliomas or gastro-intestinal stromal tumours with single *SETD2* mutations^32^,^34^. In addition, monoallelic constitutive mutations in *SETD2* were identified as the causative event in some cases of Sotos-like syndrome, an observation which offers a direct proof that monoallelic mutations in *SETD2* may have a direct pathogenic role^36^. In one case with only 3p21 heterozygous deletion, H3K36 was still detected at relatively high levels, in accordance with findings from others demonstrating that total H3K36me3 levels are not significantly impacted by monoallelic loss of *SETD2* (ref. 22).

Mutation-induced deregulation and activation of the JAK–STAT pathway, which is normally engaged by various cytokines, has emerged over the past years as a major oncogenic mechanism in several T- and NK-leukaemia and lymphoma entities^11^,^24^,^27^,^28^,^37^. Here we demonstrate that all EATL-II cases show mutations with frequent coexisting CNV or LOH in one or more genes of the JAK/STAT pathway, which is also known to regulate intraepithelial lymphocyte function^38^,^39^,^40^. Recurrent mutations of *STAT5B* in EATL-II were first reported by Kucuk *et al*. in 7/19 cases analysed in their study, and in that series occurred exclusively in tumours derived from γδ-T cells^11^. Here we found a higher incidence of mutations in *STAT5B* (17/23 cases, 74%), which was the most frequently mutated gene in the pathway. Our findings are closer to those reported very recently by Nairismagi *et al*.^10^ who found *STAT5B* mutations in 63% of the cases in a series of EATL-II from Asia. In common with the latter report, *STAT5B* mutations in our study were not restricted to tumours with γδ-TCR expression. The novel STAT5B variant (V712E) was discovered in both studies and validations performed independently by both groups convincingly point at a novel activating mutant. In addition, frequent neutral LOH at *STAT5B* locus was also observed, indicating the pivotal role of this gene in EATL-II lymphomagenesis. The frequent occurrence of *JAK3* mutations is another common finding, in one-third of the cases in the study from Asia and in about half of our cases (12/23). Moreover, we also report less frequent but recurrent mutations in *SH2B3* (20%) and in *JAK1*. We also observed that mutations affecting the JAK/STAT pathway overall are not mutually exclusive. In accordance with the observation that in other T-cell-derived neoplasms, *STAT5B* mutations tend to correlate with aggressive diseases while *STAT3* mutations occur at high frequency in indolent leukaemias^24^,^28^,^41^, interestingly, in EATL-II, the very high prevalence of *STAT5B* alterations also contrasts with the absence of *STAT3* mutations. Within our WES cohort, two patients harboured distinct mutations in GNAI2, a member of the family of G-alpha proteins involved in G-protein-coupled receptor signalling pathway, which was recently discovered^10^ as recurrently (20%) mutated in EATL-II (Supplementary Data 1)

The interplay between the JAK/STAT and the MAPK pathways is well known, as the engagement of cytokine receptors also activates the MAPK cascade^42^. The fact that we found high frequency and not mutually exclusive mutations in these two pathways suggests potential synergy in EATL-II lymphomagenesis.

The comparative analysis of the classical type of EATL (type I) was limited to a relatively small number of cases but clearly indicates a genomic profile distinct from EATL-II, since no alterations in *SETD2* were observed in any of the eight EATL-I analysed. Mutation-induced activation of the JAK/STAT pathway seems to be a feature of EATL-I as well, but relies on alterations in genes that are distinct from those most frequently involved in EATL-II: in particular, whereas no *STAT5B* mutations were identified in any of the eight EATL-I tumours tested, recurrent mutations in *JAK1* and *STAT3* were observed.

In conclusion, our study uncovers a unique genetic profile of EATL-II characterized by highly recurrent loss-of-function *SETD2* alterations and mutations affecting the JAK/STAT and MAPK pathways, supporting that a combination of epigenetic deregulation and cell signalling activation are major oncogenic events in the pathogenesis of this disease. At the same time, our findings open new avenues for the development of targeted therapies representing a highly unmet medical need for EATL-II patients. Notably, it has recently been demonstrated that H3K36me3-deficient cancers can be selectively targeted by a WEE1 inhibitor (AZD1775) (ref. 43), which might therefore constitute a new therapeutic option. Last, our analyses also substantiate that EATL-I and EATL-II are genetically different and represent distinct clinical entities.

## Methods

### Cases studied

Twenty-nine cases of EATL-II, 16 EATL-I and eight intestinal lymphomas of various histologies were retrieved from the files of the Pathology Departments of Lausanne (Switzerland), Creteil, Bordeaux, Lille and Nantes (France), and Tuebingen (Germany). The demographics of the patients and pathological features of the EATL-II cases analysed by deep sequencing are summarized in Supplementary Table 1.

The use of samples from France was approved by the Comité de Protection des Personnes – Ile-de-France IX (CPP08/009), and the use of German samples by the Ethical Committee of the University of Tuebingen (105/2013BO2). The study protocol was approved by the Commission cantonale d'éthique de la recherche sur l'être humain (Lausanne) (protocol 382/14). Informed consent could not be obtained from all subjects included in this retrospective study of a rare disease entity. Many patients were deceased at the time the study was initiated. The institutional review board (Lausanne Ethical Committee) was fully informed and consented to the study. All samples are used in accordance with the Declaration of Helsinki.

### Sample selection and preparation for sequencing

Fifteen cases of routinely processed (FFPE) surgical specimens of EATL-II were selected for availability of tumour and matching non-tumoural intestinal tissue. Areas with highest tumour cell content and those devoid of neoplastic cells were marked on HE slides by a pathologist and subsequently reported on corresponding FFPE sections stained with toluidine blue. Manual microdissection of the regions of interest was performed under a microscope, followed by genomic DNA extraction (Maxwell 16 FFPE Plus LEV DNA Purification Kit, Promega).

### Whole-exome sequencing

We developed and validated an in-house exome sequencing protocol and bioinformatic pipeline for FFPE samples by comparing WES of two cases of intestinal lymphomas for which matched frozen and FFPE blocks with paired normal and tumour tissue were available (cross-validation of FFPE and fresh-frozen mutation data—Supplementary Figs 2 and 3). Since we noticed that the amount of DNA recovered after shearing (Covaris Inc.) strongly depends on the DNA degradation status, we measured and normalized the starting material for library preparation after the sonication process. To decrease the duplication rate and thus achieve a sufficient coverage and high sequencing depth we combined the KAPA HTP library preparation kit (Kapa Biosystem) with the SureSelectXT Target Enrichment System (Agilent Technologies). Briefly, 200 ng of sheared DNA was end-repaired, extended with an A base on the 3′ end, ligated with Agilent-adaptors and pre-capture amplified with KAPA HTP library preparation kit. Exome capture was performed following the Sure Select targeted enrichment system for pair-end sequencing library protocol. Six captures were pooled further and in two lanes to a final equivalent of three exomes per lane on a HiSeq 2500 system (Illumina Inc).

### Sequence analysis

The analysis of the whole-exome sequence was based on established algorithms and pipelines according to the GATK (The Genome Analysis Toolkit) standards (Best practice variant detection with the GATK v.4, for release 2.0)^44^. Initial QC steps involved demultiplexing, quality assessment of the produced reads and adapter removal. Forward and reverse reads were aligned to the human genome (GATK repository, build 37 decoy) using BWA-MEM (v0.7.5a)^45^. BAM files were subjected to PCR duplicate removal (Picard v1.119), followed by realignment around indels and base recalibration using GATK tools (v3.2-2). Single nucleotide and Indel variant calling was performed using samtools mpileup (v1.2) and VarScan (v2.3.7)^46^ on the on-target BAM files obtained for each patient using the following parameters: samtools—minimum mapping quality=1, coefficient for downgrading mapping quality=50, minimum number gapped reads for indel candidates=3 and minimum fraction of gapped reads=0.0002; VarScan somatic algorithm—minimum variant allele frequency threshold=0.08, tumour purity 0.9, minimum read depth at a position to make a call=10 and Somatic *P* value=0.1 (Fisher's Exact Test). Raw variant calls were annotated for presence in the dbSNP and COSMIC databases as well as mutation effect on gene transcript by Annovar (v.2015-06-17)^47^. Further variant filtering was carried out in R keeping variants that showed an allele frequency ≥22% in the tumour counterpart while having allele frequency <15% in the normal as well as having at least 18 reads supporting the reference sequence and more than 5 reads supporting the variant. In addition, the differences in frequency between tumour and normal were set to be ≥22%. Variants were also excluded if part of the false positive genes reported by Fajardo *et al*.^48^ or if described to be an SNP with MAF (minimum allele frequency) <2% in either the 1000Genome project (v.Oct2014) or NIH-Exome Sequencing Project (ESP6500—European ancestry subset). MutSigCV (v1.4) was used to identify genes with a mutation frequency higher than expected^49^.

Copy number analysis was performed using the algorithm Somatic Copy Number Alteration Calling in VarScan by comparing pairs of tumour and normal samples. Raw counts were imported in R, log transformed and smoothed by computing the median using bin of 100 kb. *DNAcopy* (v1.40) and *cghMCR* (v1.24) packages were used to identify genomic regions with altered copy number. This was obtained by smoothing the data to remove single point outliers before applying a circular binary segmentation algorithm, which tests the significance of the alteration by using a hybrid approach^50^. Segments with ratio over 0.25 or under −0.25 and more than eight supporting markers were considered as ‘gain' or ‘loss', respectively. Except when indicated, all analysis was carried out using either an assortment of R system software (http://www.R-project.org, v3.1.2) packages including those of Bioconductor (v3.1) or original R code.

### Evaluation of mutation effects on SETD2

We predicted effects on stability of all six missense mutations on SETD2 using various methods^19^,^20^,^51^,^52^,^53^,^54^. With the exception of I-Mutant, which can make predictions in both sequence and structure mode, all predictions were made based on the SET and AWS (PDB-ID 4FMU) and SRI (PDB-ID 2A7O) domain structures. The SET domain structure was in a non-native, active conformation. We also created models of mutation effects on side chain positions using ANDANTE^55^, as implemented in SDM^19^.

### Pathway enrichment analysis

We assessed for enriched pathways in EATL type-II by checking if both the proportion of cases bearing at least one event (defined as either a mutation or genomic alteration) in a pathway as well as the degree by which pathways are altered in each patient, exceeded the corresponding random expectation. We used a custom signature set comprised of the MSigDB KEGG (*n*=185) and hallmark signatures (*n*=50), as well as a customized list of known epigenetic modifiers from literature^56^. Briefly, we constructed a binary event matrix, *M*_B_, comprised of all mutations and genomic events per sample. We used *M*_B_ to construct a second matrix containing the frequency of events per signature per patient (*M*_sig × patient_). To estimate the corresponding random expectation, we performed 1,000 row permutations of *M*_B_ and from which 1,000 random *M*_sig × patient, rnd_ matrices were derived. Given that rows of *M*_sig × patient_ are normally distributed, we performed a one-sided *t*-test to check if the *M*_sig × patient_ mean is greater than *M*_sig × patient, rnd._ Calculated *P* values were adjusted according to the Benjamini and Hochberg method^57^ to control for false positives.

### Sanger sequencing

Sanger sequencing was used to benchmark our variant call pipeline. Twenty-nine variants were selected among the relevant deregulated genes. DNA regions of interest were amplified using specific primers targeting exonic regions containing the variant (Supplementary Table 2). Purified PCR products were sequenced in the forward and reverse directions using ABI PRISM BigDye Terminator Cycle Sequencing Ready Reaction kits (V.3) and an ABI PRISM 3730 Genetic Analyzer (Applied Biosystems).

### Targeted deep sequencing

Targeted resequencing was used to validate some of the recurrently mutated genes identified by WES in 15 EATL-II, and to assess their mutational status in eight additional EATL-II and 8 EATL-I samples. For orthogonal validation of the 15 cases examined by WES, only tumour DNA was deep sequenced. Among the additional 16 cases, paired normal/tumour DNAs were sequenced in 10 (two EATL-II and eight EATL-I) with matching normal tissue available, and only tumour DNA was sequenced in eight EATL-II without matching normal tissue available.

The Ion AmpliSeq designer software was used to generate three highly specific panels consisting of 281 amplicons (amplicon range 125–175 bp), covering the whole coding sequence of *SETD2, CREBB* and *JAK1* and the most recurrent mutation hotspots in *JAK3* and *STAT5B* and other JAK and STAT family members described in hematopoietic or lymphoid tumours (COSMIC) and in our EATL-II cohort (Supplementary Table 3).

DNA libraries were prepared using the Ion AmpliSeq Library Kit v2 and the Ion OneTouch automated systems according to the manufacturer's instructions. Samples were sequenced on the Ion 318 Chip kit v2 (Life Technologies). The mean coverage for tumour and normal was 876 × and 803 × , respectively, with 96% of the target regions covered above 100 × .

Alignment was performed using Novoalign v3.02.07 according to the default setting. Mutations were called using Varscan v2.3.7 and annotated with ANNOVAR. SNVs and indels were filtered based on the following three conditions: (1) minimum coverage of 100; (2) minimal variant frequency 15%; and (3) *P* value (Fisher's Exact Test) for somatic variant >0.1. When matching normal DNA was available, direct comparison was performed applying the following filter for SNVs and indels call: minimum variant allele frequency threshold=0.08, tumour purity 0.9, minimum read depth at a position to make a call=10 and Somatic *P* value=0.1 (Fisher's Exact Test). Furthermore, we kept only variants that showed an allele frequency >15% in the tumour counterpart while having allele frequency <10% in the normal as well as having at least 18 reads supporting the reference sequence and more than five reads supporting the variant. In addition, the differences in frequency between tumour and normal were set to be ≥15%. Discrepancies with exome sequencing were resolved by visual inspection with IGV software to confirm or refuse the candidate alteration.

### Immunohistochemistry

IHC staining was done on 4 μm FFPE sections from native blocks and from a tissue microarray comprising cores representative of a subset of EATL-II cases. Reactive tonsils were used as external controls for all staining. Briefly, after deparaffinization, antigen retrieval was performed in Tris/EDTA pH 9.0 in a pressure cooker respectively 1.30 min for Histone H3, H3K36me3 and H3K36me2 and 5 min for SETD2. Slides were incubated with Histone H3 (Abcam, ab1791, dilution 1:1,000), H3K36me3 (Abcam, ab9050, dilution 1:2,000) or H3K36me2 (Abcam, ab 9049, 1: 4,000) or SETD2 (Sigma, HPA042451, 1:400) antibodies for 1 h at room temperature. Antibody detection was performed with diaminobenzidine detection system (Dako), according to the manufacturer's instructions. For SETD2 we used a C-terminal *SETD2* antibody that would not detect *SETD2* affected by protein truncations distal to the reported mutations. Two pathologists independently performed quantification of H3K36me3, H3K36me2 and SETD2, blinded to the *SETD2* genotype. The individual scores were overall concordant and the cases were reviewed for consensus final scoring. Specific attention was paid to the cases that showed discordant genotype/phenotype.

A semi-quantitative scoring scale was applied, based on both the extent and intensity of the staining as previously published^58^. Staining of <10% of stained tumour cells was considered negative (score 0); scores 1, 2, 3 and 4 corresponded to positivity in 10–25%, 26–50%, 51–75% and 76–100% of tumour cells, respectively. Negative stainings with no internal positive control were not interpreted. For staining intensity, scores 1, 2 and 3 were attributed to weak, moderate and strong intensities, respectively; undetectable staining was scored 0. To provide a common global scale, the extent score and the intensity score were multiplied with a scoring scale ranging from 0 to 12. Furthermore, the ratio of H3K36me3 score to that of H3K36me2 score was calculated to normalize the level of trimethylation and correct for difference across different samples with heterogeneous bimethylation levels.

### FISH assays

To validate CNV data from WES, we developed original FISH probes designed to identify deletions of the *SETD2* locus at 3p21.31. Bacterial artificial chromosome (BAC) clones were chosen using the UCSC (University of California Santa Cruz) Genome Browser website (http://genome.ucsc.edu, February 2009 assembly) and purchased from BACPAC Resources. We selected two BAC clones hybridizing to the *SETD2* locus (target probes at 3p21.31: RP11-425J9 and RP11-650F17, GenBank references AC094020.2 and AQ463932.1/AQ511510.1, respectively) and two BAC clones hybridizing to a preserved region on chromosome 3 (control probe at 3p25: RP11-266J6 and RP11-485N3, GenBank references AC011610.11/AC022382.4 and AQ633871.1/AQ633873.1, respectively), as illustrated in Supplementary Fig. 3. Following overnight culture, BAC DNA was extracted (Qiagen Plasmid Maxi Kit, Qiagen) and fluorescently labelled by nick translation (Nick Translation Kit, Abbott Molecular). BACs constituting the target probes were labelled with SpectrumOrange-dUTP and those forming the control probe with SpectrumGreen-dUTP (Abbott Molecular), and resuspended at 100 ng μl^−1^ in LSI/WCP Hybridation Buffer (Abbott Molecular). Each BAC was tested separately on metaphase spreads from normal peripheral blood, in conjunction with a centromeric probe for chromosome 3 (Vysis CEP3 (D3Z1) SpectrumOrange Probe, Abbott Molecular), to verify hybridization to the expected chromosomal region and rule out any cross-hybridization.

FISH labelling was performed using the Histology FISH Accessory Kit (Dako) according to the manufacturer's protocol, with minor modifications. After dewaxing and rehydration, 4-μm-thick tissue sections were heated in pre-treatment solution (Dako) for 10 min in a water-bath at 95 °C. Slides were then digested with pepsin (Dako) for 13 min at 37 °C on an open preheated hybridizer (Dako). Following dehydration, FISH probes were placed onto the section and covered with sealed coverslip. Codenaturation (5 min at 73 °C) and hybridization (overnight at 37 °C) of probes and sample were successively carried out in a closed humidified hybridizer. After coverslip removal, probe excess was washed in 2 × SSC/0.3% Tween20 for 2 min at 73 °C followed by 2 × SSC/0.1% Tween20 for 2 min at room temperature. Finally, slides were dehydrated and mounted with DAPI I counterstain (Abbott Molecular) and maintained at 4 °C in the dark.

Labelled slides were analysed with a Zeiss AxioImager Z2 fluorescence microscope equipped with specific filters for FITC, SpectrumOrange, DAPI, and double and triple band-pass filters. Hybridization signals were examined with a Plan-APOCHROMAT × 63 oil immersion objective. Images were captured using ISIS digital image analysis system version 5.5 (Metasystems).

To establish the cutoff for *SETD2* locus deletion, we recorded the hybridization patterns of at least 200 nuclei in five negative control samples (reactive tonsils from healthy individuals). A nucleus was considered deleted for *SETD2* locus if the ratio between the orange signals (target probe) and the green signals (control probe) was ≤0.5. We thereby obtained a cutoff value of 11.2%, computed as the mean percentage of nuclei with *SETD2* locus deletion (false-positive findings) plus three s.d.'s, in line with published recommendations.

The evaluation of *SETD2* locus in tumour samples was carried out in the regions with highest tumour cell content, selected using the DAPI filter under the guidance of corresponding HE slides. For each case, the hybridization patterns of at least 100 tumour nuclei were recorded. If the percentage of nuclei with *SETD2* locus deletion was above the cutoff value of 11.2%, the case was considered deleted.

### STAT5B mutagenesis

An expression plasmid pCMV6-Entry containing the wild-type sequencing of STAT5B (OriGene) was used as a template for site-directed mutagenesis to generate STAT5B mutants for functional study. STAT55-N642H and STAT5B-V712E mutants were generated using the Quick-change Site-directed Mutagenesis kit (Agilent Technology), according to the manufacturer's instructions. The primers used for site direct mutagenesis are as follows:

STAT5B-N642H-F: 5′-GGTGGTAAAAGGCATCAGATGCCAAAACATTCTTTCCTGAG-3′

STAT5B-N642H-R:5′-CTCAGGAAAGAATGTTTTGGCATCTGATGCCTTTTACCACC-3′

STAT5B-V712E-F: 5′-GTGGTCCCTGAGTTTGAGAACGCATCTGCAGAT-3′

STAT5B-V712E-R: 5′-ATCTGCAGATGCGTTCTCAAACTCAGGGACCAC-3′

Full sequencing of the inserts was performed to confirm the presence of the introduced mutations and to ensure that no additional variants were added during the mutagenesis reaction.

After mutagenesis STAT5B-WT, -N642H and -V712E cDNAs were PCR-cloned into the multiple cloning site of the pRRL-PGK-T2A-GFP lentiviral expression vector (kindly provided by Dr George Coukos) using BclI and NheI restriction sites.

### Cell line

Hela cell line from American Type Culture Collection (ATCC) was maintained in DMEM (Sigma-Aldrich) containing 10% FBS and supplemented with 2 mM L-glutamine and 1% Penicillin–Streptomycin, at 37 °C and 5% CO_2_.

### Transient transfection and luciferase assay

Eighty per cent of confluent Hela cells were transiently transected with pRRL-PGK-T2A-GFP–STAT5B/WT, /N642H, /V712E and with empty vector respectively using Lipofectamine 2000 (Thermo Fisher) according to manufacturer's instructions. Cells were simultaneously co-transfected with pGL4.52 vector, a luciferase report construct with a STAT5 responsive element luc2P/STAT5 RE/Hygro (Promega), and with a renilla luciferase pRL-TK plasmid (Promega). After 48 h transfection, The Dual-Luciferase Reporter Assay System (Promega) was used to determine luciferase activity according to the manufacturer's recommendations. Firefly and renilla luciferase activities were measured sequentially; results were expressed as ratios of firefly luciferase activity over renilla luciferase activity. The experiments were performed in triplicate.

### Western blotting

Hela cells were lysated after 48 h transfection with pRRL-PGK-T2AGFP–STAT5B WT and mutant constructs. Non-transfected cell line and cell transfected with an empty vector were used as internal control for the reaction. Standard western blot analysis was carried out. RIPA buffer supplemented with protease inhibitors cocktail (Sigma Aldrich) and phosphates inhibitor cocktail (Sigma Aldrich) was used for protein extraction. Proteins were transferred to a polyvinylidene difluoride membrane, after which the membrane was blocked with Super Block (Thermo Fisher) blocking buffer for 1 h. Primary antibodies for STAT5B were obtained from Cell Signal Technology: STAT5B Rabbit mAb#9363 dilution 1:1,000 in 5% milk and Phospho-Stat5 (Tyr694) (D47E7) Rabbit mAb #4322 dilution 1:500 in Super block. Monoclonal Anti-Actin (Clone AC-40) and anti-GFP (clone GFP-20) antibodies were purchased from Sigma Aldrich and used to a working dilution of 1:1,000. Membranes were incubated overnight at +4 °C. Uncropped representative WB images are shown in Supplementary Fig. 11.

### LOH on chromosome 17

To validate LOH observed on chromosome 17, we performed a TaqMan SNP genotyping assay using QuantStudio 3D digital PCR. Starting from our WES data, we selected the SNP rs1126821 (assay ID: C_153647_20) which was observed to be polymorphic in five samples (four with LOH and one with no alterations in that locus) and at ∼439 kb upstream to the genomic locus for *STAT5B* gene. All the assays were performed following manufacturer's instructions and analysed with QuantStudio 3D Analysis Suite Cloud Software.

### Data availability

The VCF files containing the somatic variants of the samples analysed by whole-exome sequence have been deposited in the European Genome Archive (EGA) under accession code EGAS00001001879. SET and AWS domain structures (PDB-ID 4FMU) and the SRI domain structure (PDB-ID 2A7O) were obtained from PDB. All other data are available in the paper and Supplementary Information files, or available from the author upon request.

## Additional information

**How to cite this article:** Roberti, A. *et al*. Type II enteropathy-associated T-cell lymphoma features a unique genomic profile with highly recurrent *SETD2* alterations. *Nat. Commun.* 7:12602 doi: 10.1038/ncomms12602 (2016).

## Supplementary Material

Supplementary Information Supplementary Figures 1-11 and Supplementary Tables 1-6.

Supplementary Data 1 Somatic non-silent mutations identified by WES. Variants that were validated by Sanger and/or targeted deep sequencing are indicated in boldface. Variants that were not validated are marked by an asterisk and were excluded from mutational landscape.

Supplementary Data 2 Pathways enriched for mutations in EATL-II ordered by significance (adj.p.value <=0.05). The size of each pathway, together with the number of mutated elements are indicated. The identities of the mutated genes in the pathways are indicated in the last column.

Supplementary Data 3 Summary of copy number variations (gains/losses; amplifications/deletions) in the 15 EATL-II cases subject to WES. Mutated genes for each reported locus are indicated in the last column.

## Figures and Tables

**Table 1:**
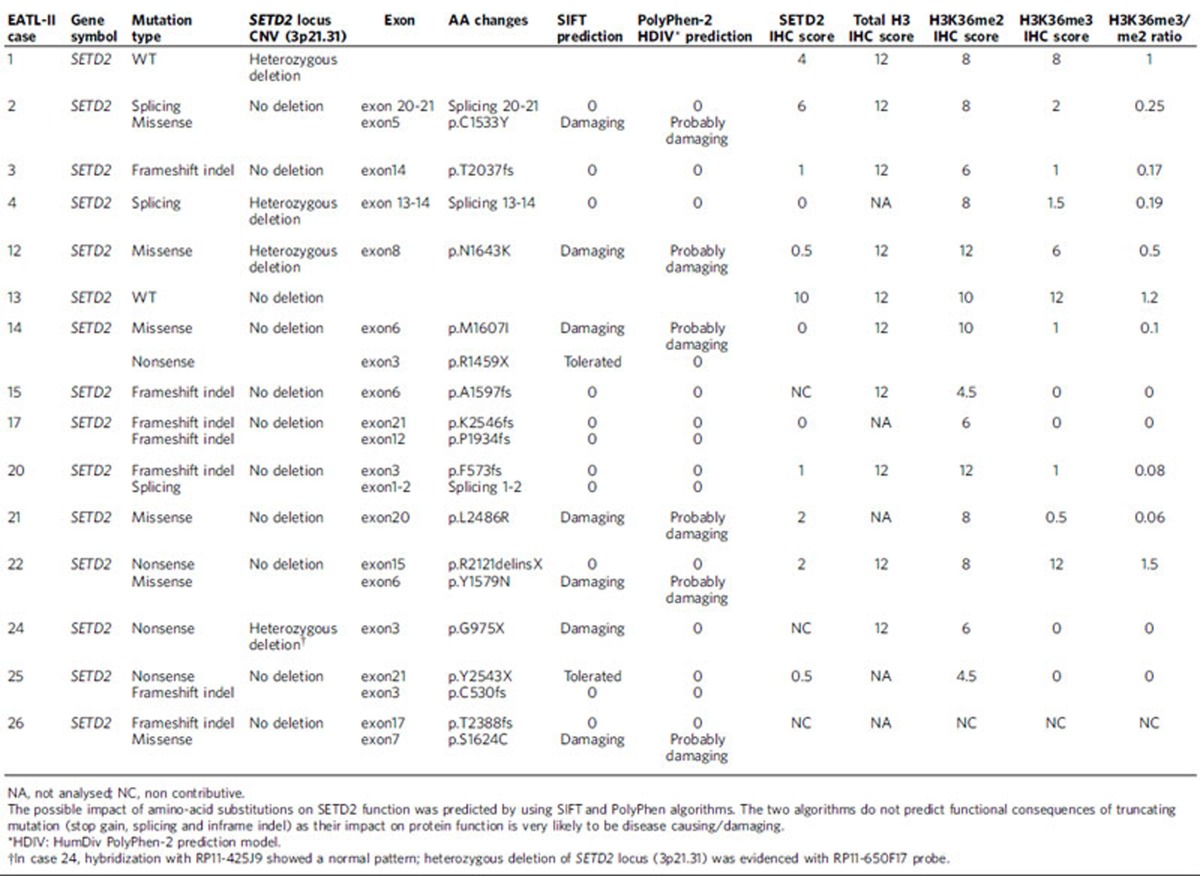
Summary of *SETD2* genomic status (mutations and copy number) with phenotypic correlation (expression of SETD2 and H3K36 methylation) in the 15 EATL-II analysed by WES.
