# Supplementary material for: Type II enteropathy-associated T-cell lymphoma features a unique genomic profile with highly recurrent SETD2 alterations
Source: Nat Commun. 2016 Sep 7;7:12602. doi: 10.1038/ncomms12602 (PMC5023950; doi:10.1038/ncomms12602)
Supplement: Supplementary Information — Supplementary Figures 1-11 and Supplementary Tables 1-6. [file ncomms12602-s1.pdf]

Supplementary Information

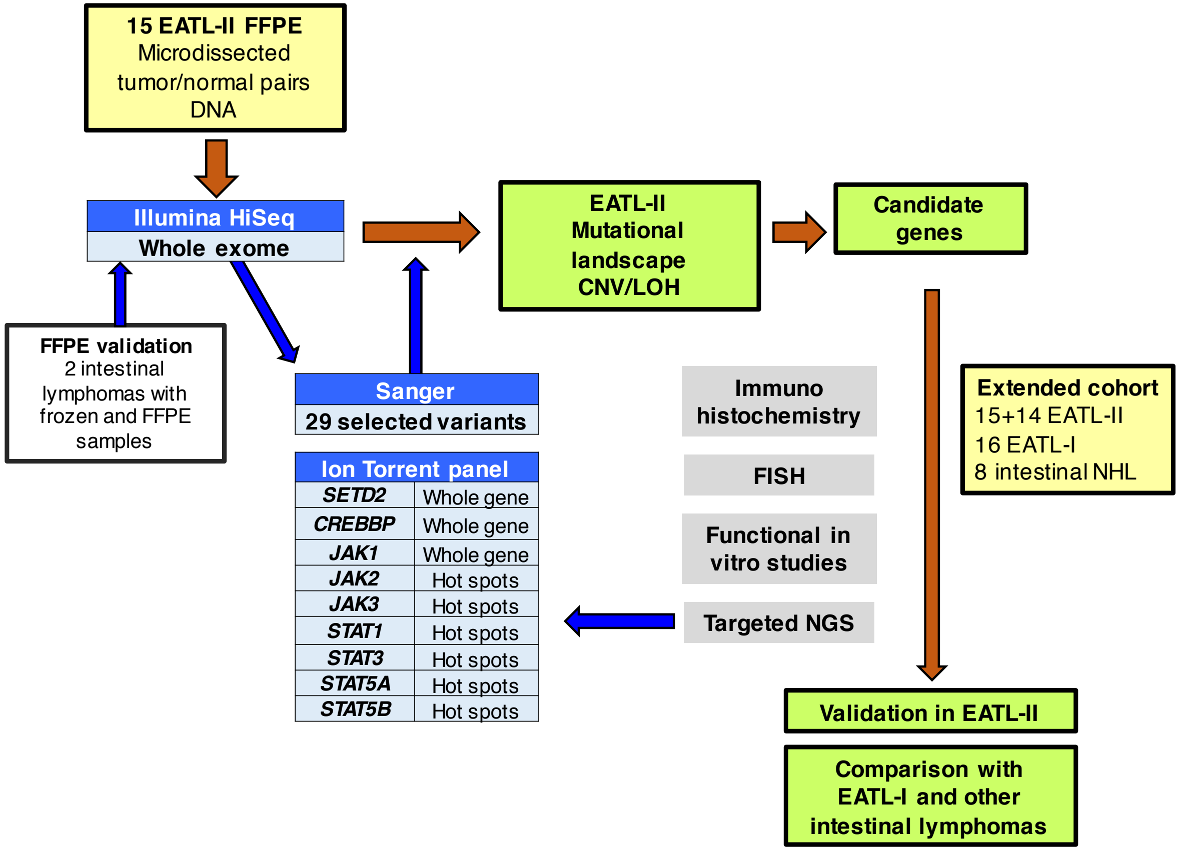

Supplementary Figure 1. Study design and workflow analysis. We adapted a sequencing protocol to be suitable to formalin-fixed paraffin-embedded (FFPE) tissues (**Supplementary Figures 7** and **8**), and performed whole-exome sequencing (WES) of 15 paired normal and tumor DNA samples microdissected from FFPE EATL-II specimens. A subset of the mutations identified by WES was validated by Sanger and/or targeted deep sequencing. An extended cohort comprising 14 additional EATL-II, 16 EATL-I and 8 other intestinal lymphomas of various histotypes, was used for validation of the main candidate gene and relied upon immunohistochemistry, FISH and targeted sequencing. Functional studies assessing the activity of variants were performed in vitro with cell line.

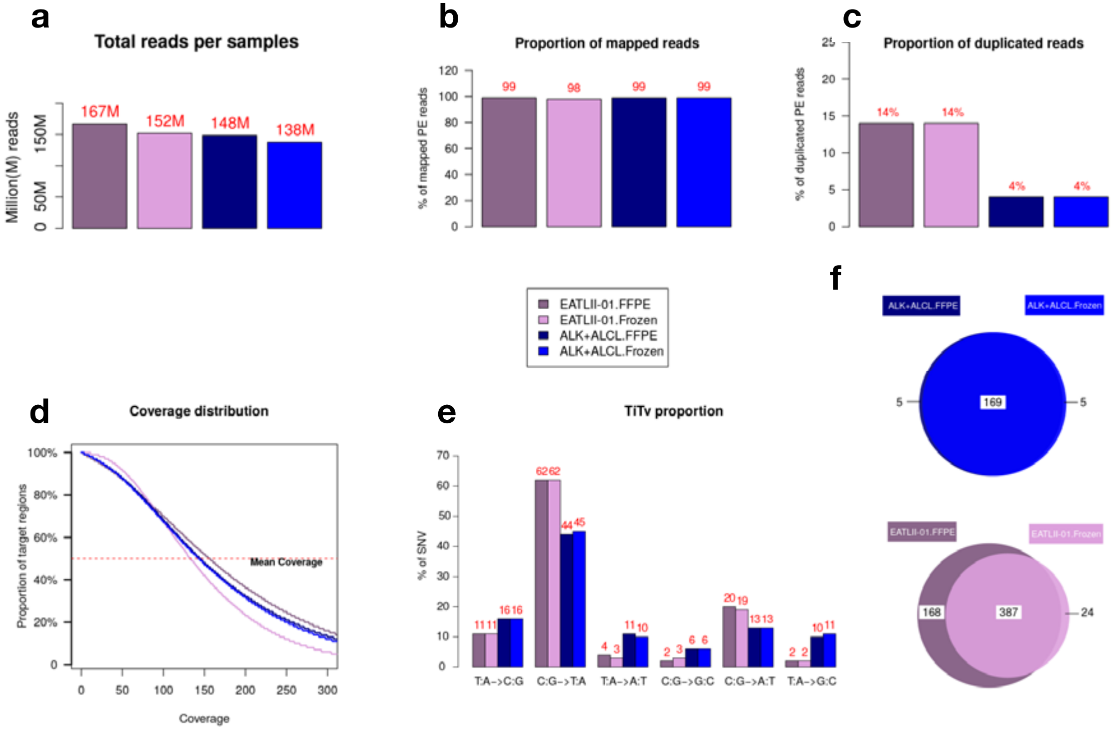

Supplementary Figure 2. Comparison of mutation data generated from WES of frozen and formalin-fixed paraffin-embedded samples. Starting from the notion that fresh-frozen (FF) material represents a benchmark for WES technique, we wanted to assess the quality of our WES protocol and bioinformatic pipeline on formalin-fixed, paraffin-embedded (FFPE) samples by comparing two paired normal/tumor (one case of EATL-II (case 1) and one case of ALK-positive anaplastic large cell lymphoma (ALCL) of the intestines) for which FF and FFPE tissue samples were available. Number of total reads (a) as well as the proportion of mapped reads (b) was comparable between FF and FFPE paired samples. The degree of duplication was overall low and no differences were observed between FF and FFPE (c), this generated an equal coverage rate among samples (d), indicating that this protocol can overcame the challenging and variability in data quality associated with DNA degradation in FFPE samples. Also the spectrum of mutations was similar (e) in particular FFPE samples

did not` show a higher rate of C>T (and G>A) sequence artifacts. Moreover we observed an overlap of non-silent mutations between FF and FFPE in the ALK-positive ALCL and EATLII-01 of 97% and 94% respectively (f).

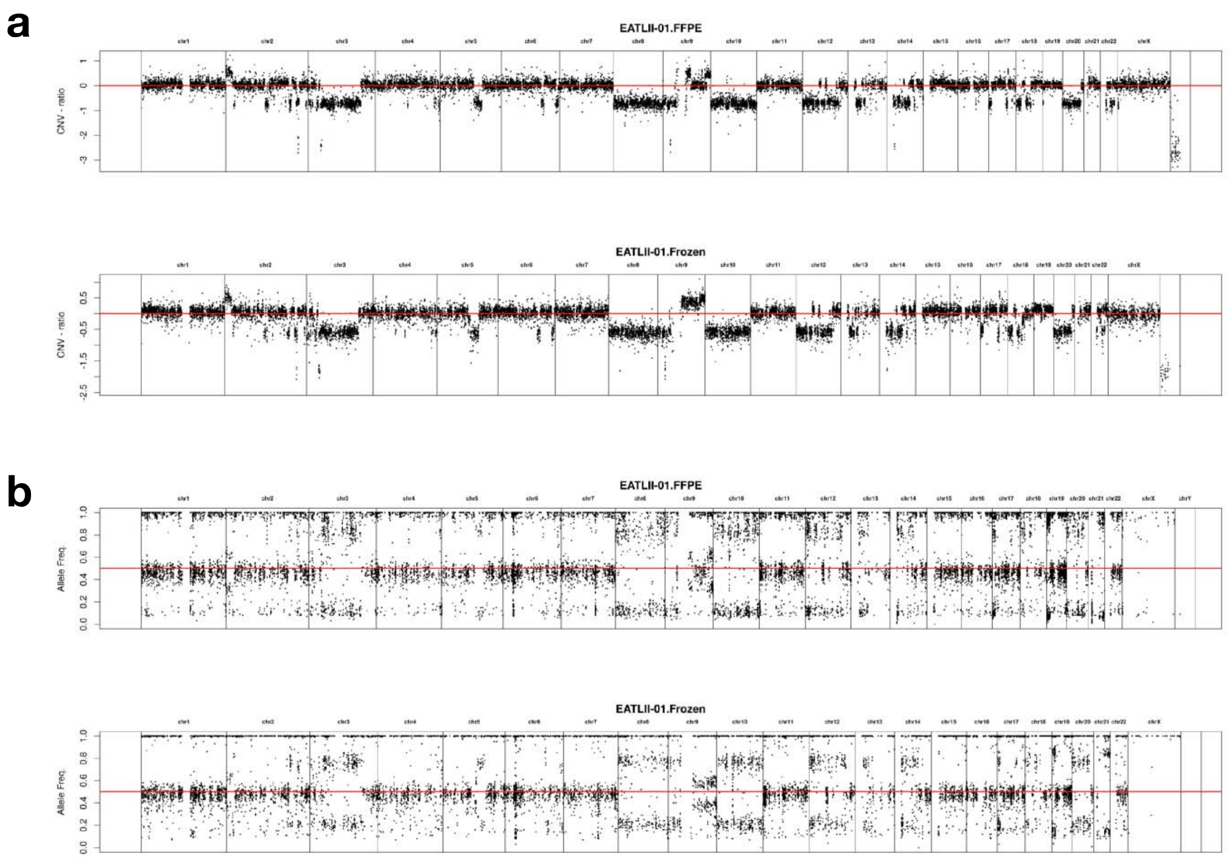

Supplementary Figure 3. Comparison of copy number variation data generated from WES of frozen and formalin-fixed paraffin-embedded samples. Copy number variation (CNV) (a) and allele frequency profiles (b) of the FF and FFPE EATLII-01 tumor specimens were also very much alike, supporting the notion that our WES protocol on FFPE material was providing results widely similar to the one obtained by FF material.

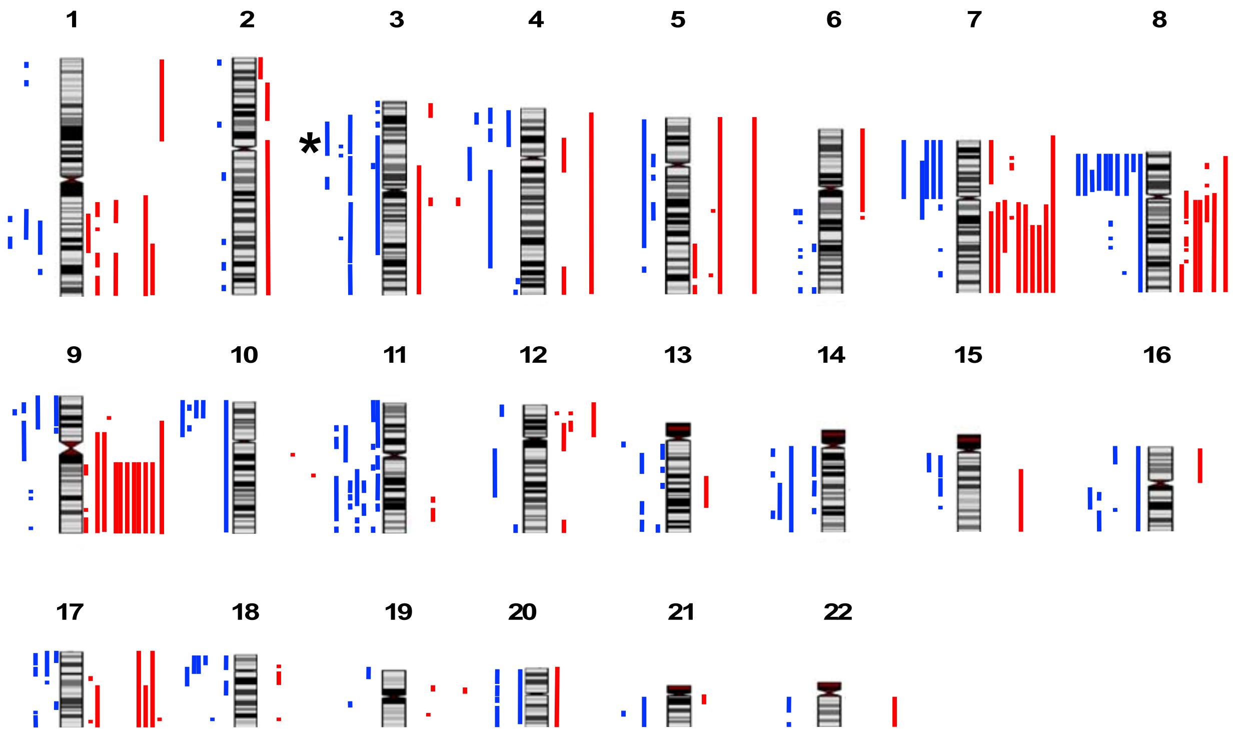

Supplementary Figure 4. Human chromosomal ideograms showing the areas of genetic gain and loss identified by whole-exome sequencing in 15 EATL-II samples. Red bars to the right of the chromosomes represent areas of gain and blue lines on the left side of the ideogram indicate areas of loss. Loss of *SETD2* at 3p.21in 4 EATL-II patients is showed as an asterisk.

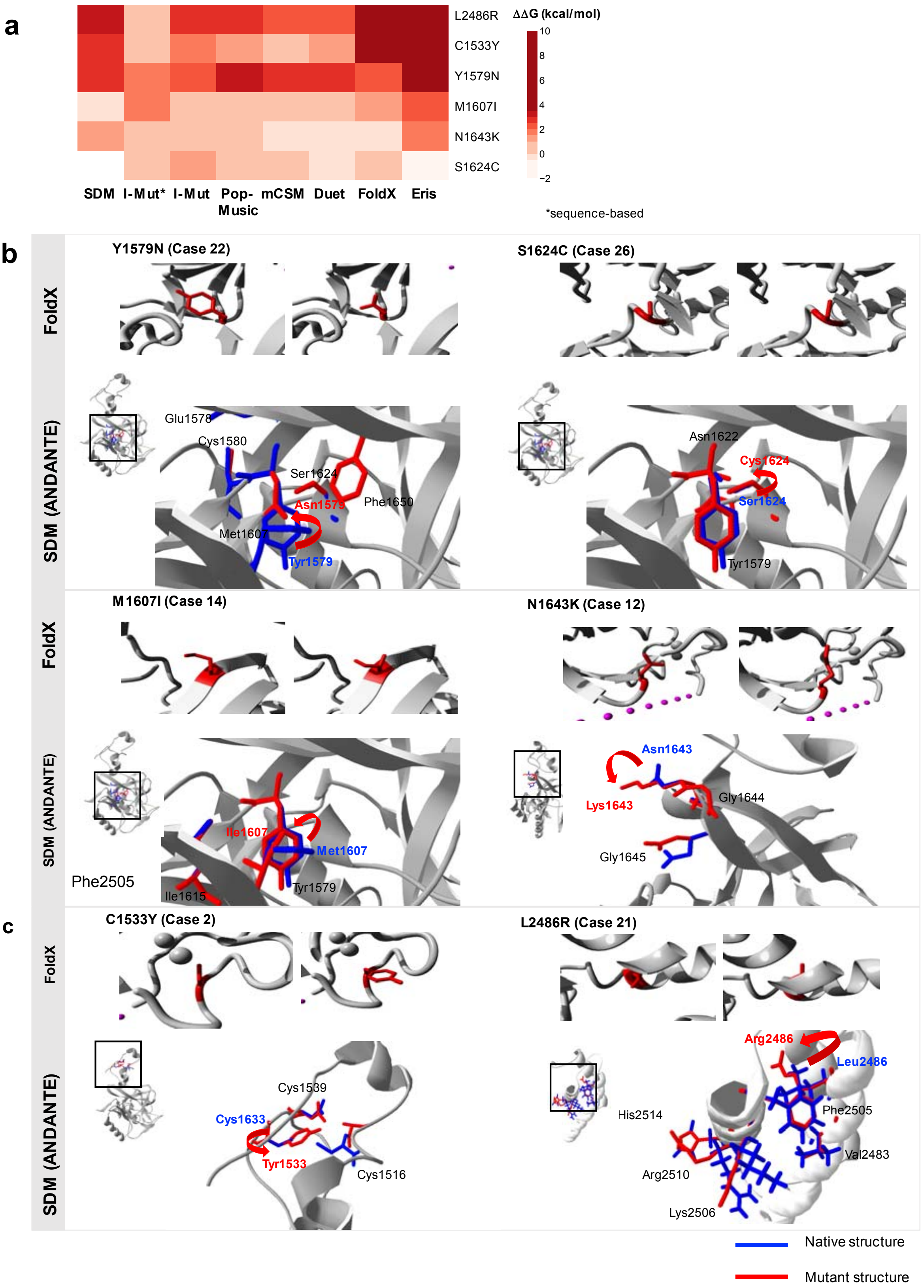

Supplementary Figure 5. (a) Predicted effects of six point mutations on SETD2 stability. Except for SDM and Eris results for S1624C and SDM results for M1607I, all SET (**b**), AWS

(c, left panel) and SRI domain **(c, right panel)** are consistently predicted to be destabilizing by various sequence- and structure-based predictors. S1624C, despite being predicted stabilizing, is nonetheless predicted to cause protein malfunction and disease by SDM. All structure-based predictions were based on PDB structures 4FMU (SET and AWS domains) and 2A7O (SRI domain). Models of SET domain mutants from FoldX or Andante illustrate predicted changes in the domain structure. Note that mutated positions in the SET domain, 1579, 1607 and 1624, are spatially proximal and are all located within the SET binding pocket. All neighboring residues within a 3.5 Å (SET and AWS domains) or that are known to impact binding (SRI domain) are shown. Except for Y1579N (Case 22), all mutations have been functionally confirmed by IHC as inactivating.

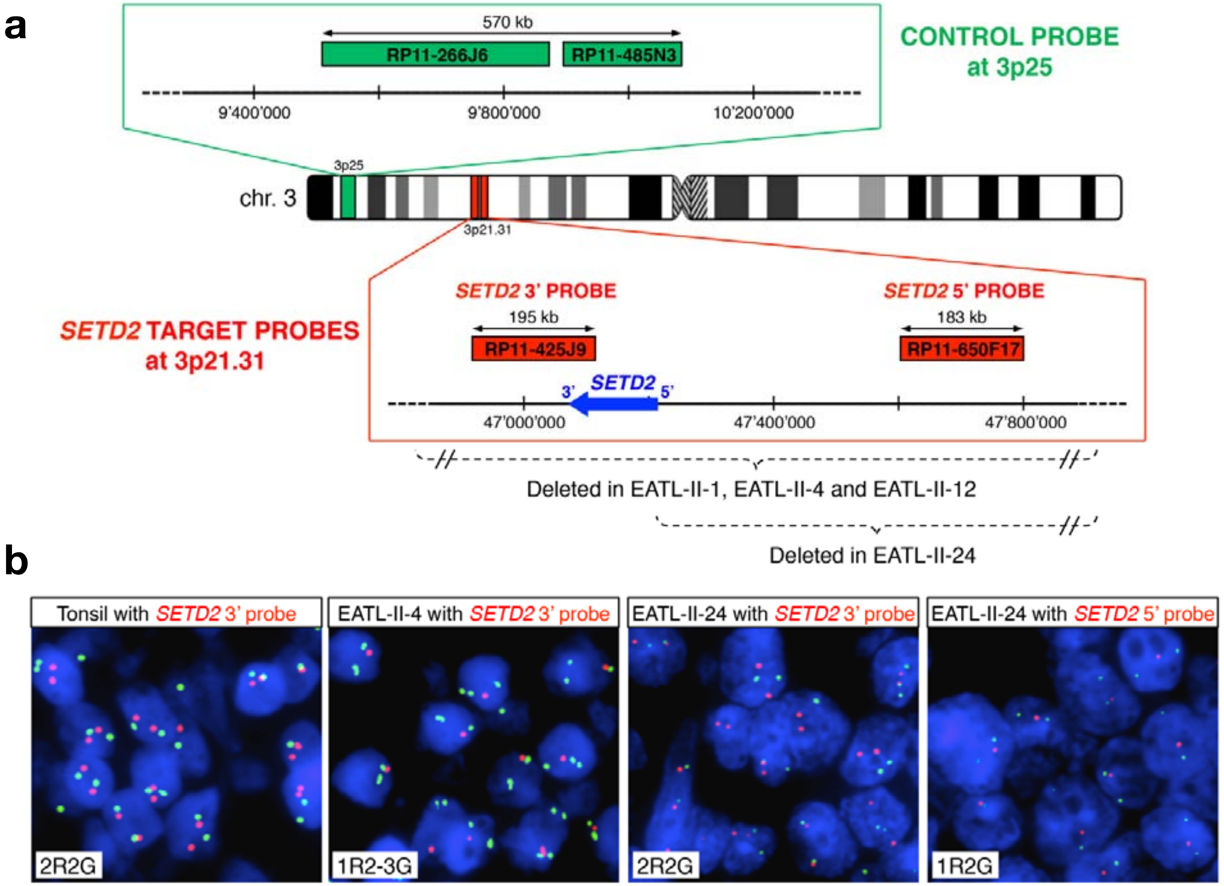

Supplementary Figure 6. Design for fluorescence in situ hybridization (FISH) analysis of *SETD2* locus deletions at 3p21.31 (a) Two bacterial artificial chromosome (BAC) clones hybridizing to the *SETD2* locus at 3p21.31 were labelled in red, forming two distinct *SETD2* target probes: *SETD2* 3′ probe, composed of BAC RP11-425J9, complementary to the 3′ end of the *SETD2* gene that was deleted in EATL-

II cases 1, 4 and 12 according to CNV analysis ; and *SETD2* 5′ probe, constituted of BAC RP11-650F17, hybridizing to a region 5′ to the *SETD2* gene, which was deleted in case EATL-II-24 (in addition to cases 1, 4 and 12). Another set of two BAC clones hybridizing to the 3p25 region (preserved in all the samples to be tested according to CNV analysis) was labelled in green, building up together the 3p25 control probe (RP11-266J6 and RP11-485N3). Representative *SETD2* FISH images of a reactive tonsil and of cases EATL-II-4 and EATL-II-24 (original magnification x630; cases EATL-II-1 and EATL-II-12 are illustrated in Figure 2c) **(b)** Far left - Reactive tonsil used as a negative control, hybridized with *SETD2* 3′ probe, revealing a normal diploid hybridization pattern, i.e., two red *SETD2* signals and two green control signals per nucleus (an identical pattern was observed with *SETD2* 5′ probe, not shown). Middle left - Case EATL-II-4 analyzed with *SETD2* 3′ probe, showing a heterozygous *SETD2* locus deletion (one red *SETD2* signal and two-three green control signals per nucleus). Middle right and far right - Case EATL-II-24 analyzed separately with *SETD2* 3′ probe (middle right) and *SETD2* 5′ probe (far right): as expected, no deletion was observed with *SETD2* 3′ probe (tow red *SETD2* signals and two green control signals per nucleus), whereas a heterozygous *SETD2* locus deletion was demonstrated with *SETD2* 5′ probe (two red *SETD2* signal and two green control signals per nucleus).

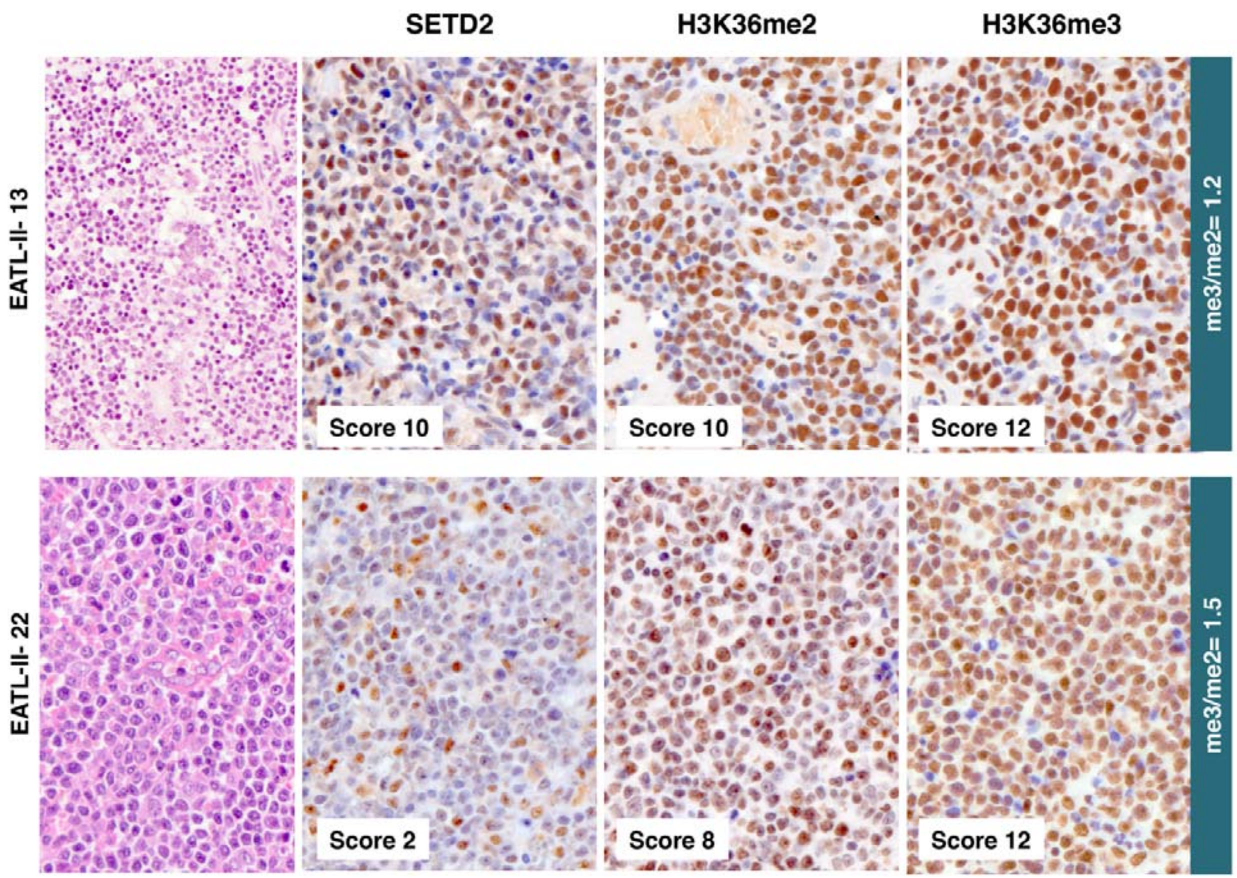

Supplementary Figure 7. Morphology (hematoxylin and eosin, original magnification x400) and immunohistochemical stainings for SETD2, H3K36me2 and H3K36me3 (immunoperoxidase, original magnification x400) in two EATL-II cases with preserved H3K36 trimethylation (EATL-II-13: no *SETD2* mutations and no 3p21 loss; EATL-II-22: biallelic *SETD2* mutations).

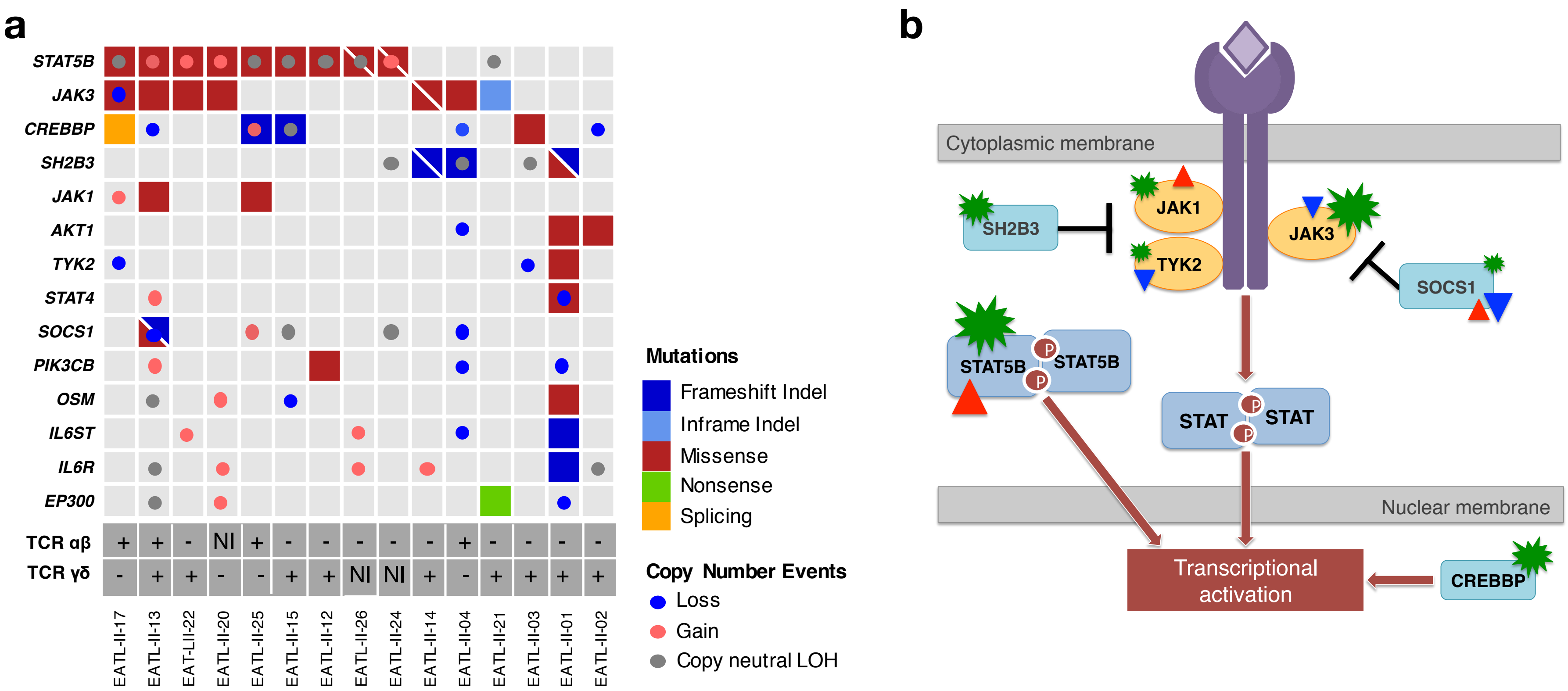

Supplementary Figure 8. Mutations in genes of the JAK/STAT pathway (KEGG pathway ID: hsa04630) are represented for each of the 15 EATL-II samples analyzed and arranged by decreasing mutation frequency **(a)**. Biallelic or double mutations are represented as triangles, single mutations as full colored squares. Colored dots indicate copy number variations (gains, pink and losses, blue) or loss of heterozygozity (grey) at the corresponding loci. Immunohistochemical staining results for the expression of the αβ and γδ isoforms of the T-cell receptor (TCR) are reported. Diagram showing the JAK/STAT pathway components most frequently mutated in EATL-II patients **(b)**. Mutations are shown as green stars, copy gains and losses are represented respectively as red and blue triangles, sized according to their frequency in our cohort of 15 cases.

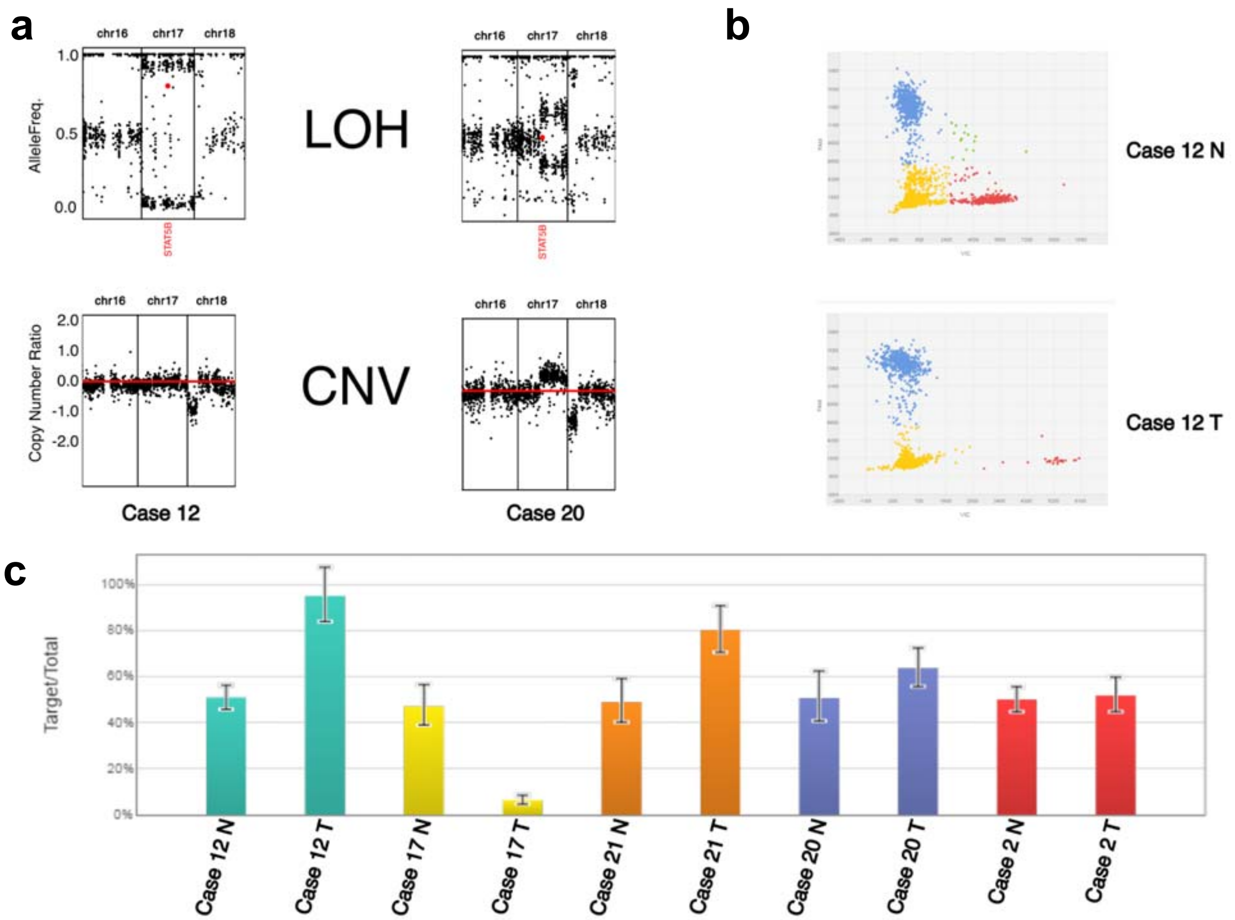

Supplementary Figure 9. Loss-of-heterozygosity (LOH) in all or part of chr17 was observed in 9 out 15 EATL-II cases analyzed by WGS. Of these 6 events were associated with a neutral-copy-number status. **(a)** Examples of neutral LOH (Case 12) and LOH associated with 1 copy number gain (Case 20). Top graphs show allele frequency distribution for chromosome 16, 17 and 18, while the bottom graphs show

the associated CNVs for the same genomic regions. **(b)** Example of digital PCR results performed using TaqMan SNP genotyping assay and QuantStudio 3D machine on Case 12. Blue and red dots represent wells in which PCR products were generated starting from the two alternative TaqMan probes for the SNP rs1126821, while yellow dots represent unamplified wells. The top graph shows the results obtained from normal DNA, in which both nucleotide of the SNP are equally represented. On the contrary, in the bottom graph tumor component showed a strong bias versus one of the probe, indicating a loss of the other allele. **(c)** The results of all the assays performed are summarized as ratio between the two probes (allele frequency) in a barplots. Case 12, 17 and 21 were confirmed to have LOH associated with absence of CNV events (WGS data). Case 20 showed also LOH but associated with a gain of one copy (WGS data). Case 2 was chosen as negative control. All experiments were done in duplicates.

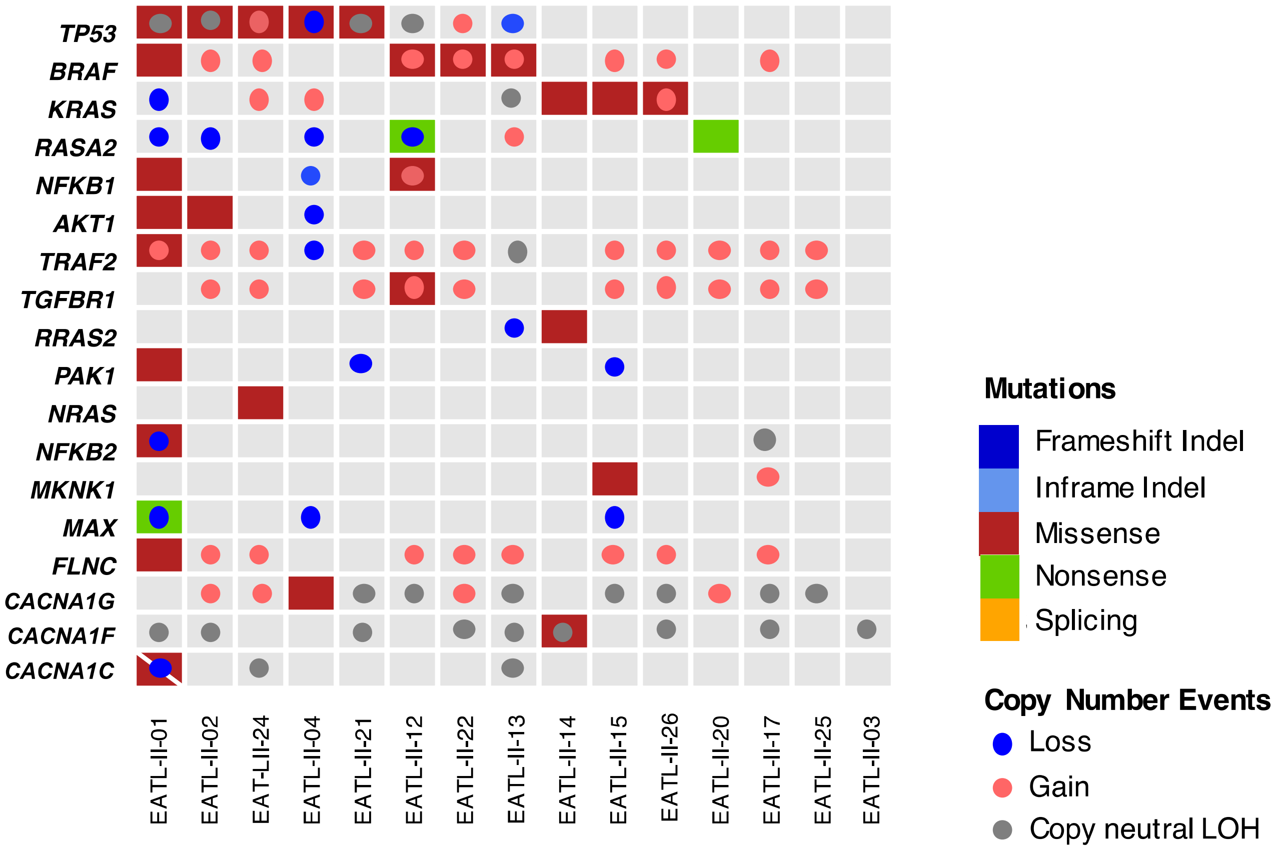

Supplementary Figure 10. Mutations in genes of the MAPK pathway (canonical pathway and p53 signalling pathway branch, KEGG pathway ID: hsa04010) are represented for each of the 15 EATL-II samples analyzed and arranged by decreasing mutation frequency. Biallelic or double mutations are represented as triangles, single mutations as full colored squares. Colored dots indicate copy number variations (gains, pink and losses, blue) or loss of heterozygozity (grey) at the corresponding loci.

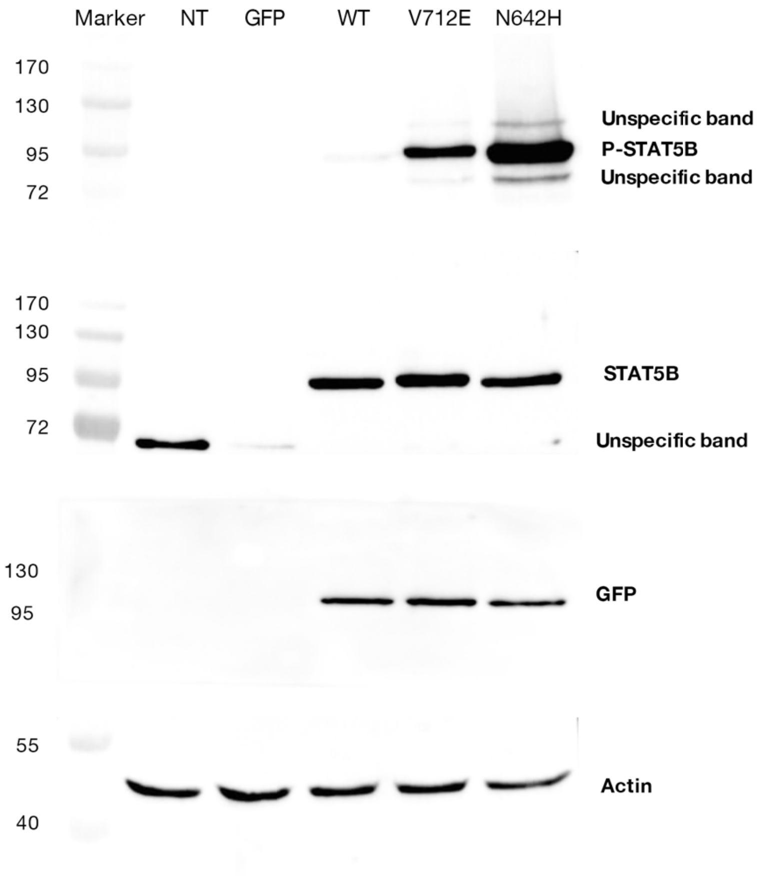

Supplementary Figure 11. Raw western blot profiles corresponding with Figure 3c.

Supplementary Table 1: Summary of clinicopathological features of the EATL-II cases subject to sequencing. Cases 28 and 30 were subject to targeted deep sequencing only; all other cases were subject to whole-exome and targeted deep sequencing.

Case	SEX	LOCALIZATION	PLEOMORPHISM	CYTOLOGY	EPITHELIOTROPISM	CD2	CD3	CD4	CD5	CD7	CD8	CD56	CD57	TIA1	GRANZYME B	PERFORINE	CD20	CD79a	MIB1	TCR BETAF1	TCR GAMMA	CD103	WES	TARGETED SEQUENCING
EATL-II-1	M 73	small intestines	Monomorphic	Small to medium	Present	pos	pos	neg	neg	pos	pos	pos	neg	pos	pos	neg	neg	neg	85%	neg	pos	pos	yes	yes
EATL-II-2	M 45	small intestines	Pleomorphic	Medium to large	Not seen	neg	pos	neg	neg	pos	neg/pos	pos	neg	pos	pos	neg/pos	neg	ND	>95%	neg	pos	neg	yes	yes
EATL-II-3	M 49	small intestines	Monomorphic	Small to medium	Present	neg	pos	neg	neg	pos	pos	pos/neg	neg	pos	pos	neg	neg	neg	20%	neg	pos	pos/neg	yes	yes
EATL-II-4	F 81	small intestines	Monomorphic	Small to medium	Present	pos	pos	neg	neg	pos	pos	pos/neg	neg	pos	neg/pos	neg	neg	ND	80%	pos wk	neg	pos/neg	yes	yes
EATL-II-12	F 73	small intestines	Monomorphic	Small to medium	Present	pos	pos	neg	neg	pos	pos	pos	neg	pos	neg/pos	neg/pos	neg	neg*	80%	neg	pos	pos/neg	yes	yes
EATL-II-13	M 54	small intestines	Monomorphic	Small to medium	Present	neg	pos	neg	neg	pos	neg	neg	neg	pos/neg	neg	neg	neg	neg	60%	pos	pos	pos	yes	yes
EATL-II-14	F 67	small intestines	Monomorphic	Small to medium	Present	neg	pos	neg	neg	pos	pos	pos	neg	pos	neg/pos	neg	neg	neg	80%	neg	pos	neg/pos	yes	yes
EATL-II-15	F 76	small intestines	Monomorphic	Small to medium	Not evaluable	neg/pos	pos	neg	neg	pos	pos	pos	neg	pos	pos/neg	neg	neg	neg	60%	neg	pos	neg/pos	yes	yes
EATL-II-17	M77	small intestines	Monomorphic	Small to medium	Present	neg	pos	neg	neg	pos	pos/weak	pos	neg	pos	pos/neg	pos	neg	neg	80%	pos	neg	pos	yes	yes
EATL-II-20	M 47	small intestines	Monomorphic	Small to medium	Not evaluable	pos	pos	neg	neg	pos	pos/neg	pos	neg	pos	pos	neg/pos	neg	neg	85%	NI	neg	pos/neg	yes	yes
EATL-II-21	M 64	small intestines	Monomorphic	Medium	Present	neg	pos weak	neg	neg	pos	neg/pos	pos	neg	pos	pos/neg	pos	neg	neg	90%	neg	pos	neg	yes	yes
EATL-II-22	M 51	small intestines	Monomorphic	Medium	Present	neg	pos	neg	neg	pos	neg/pos	pos	neg	pos	neg/pos	pos/neg	neg	pos/neg	70%	neg	pos	neg/pos	yes	yes
EATL-II-24	M 66	small intestines	Monomorphic	Medium to large	Not evaluable	neg	pos	neg	neg	pos	pos	pos	neg	pos	neg/pos	pos	neg	neg/pos	50%	neg	NI	neg/pos	yes	yes
EATL-II-25	F 64	small intestines	Monomorphic	Medium	Present	neg	pos	neg	neg	pos	pos	neg	neg	pos	NI	pos	neg	neg	80%	pos	neg	pos	yes	yes
EATL-II-26	F 76	colon	Monomorphic	Small to medium	Present	neg	pos	neg	neg	pos	neg	neg	neg	pos	neg/pos	neg/pos	neg/pos	neg	20-30%	neg	NI	pos	yes	yes
EATL-II-28	M62	small intestines	Monomorphic	Large	Not seen	neg	pos	neg	neg	pos	neg	pos/weak	ND	ND	pos	ND	ND	ND	95%	neg	pos	pos	no	yes
EATL-II-30	F47	small intestines	Monomorphic	Medium to large	Not seen	neg	pos	neg	neg	pos	neg	neg/pos	neg	pos	neg/pos	neg	neg	ND	80%	neg	neg	neg/pos	no	yes

NI= not interpretable
ND= not done

Supplementary Table 2: Primers used for Sanger sequencing validation of mutations discovered by WES. 26 primer pairs were used to validate 29 different variants that occurred in one to 5 cases each. The 24 variants that were confirmed by Sanger sequencing are indicated in boldface. Five variants were not confirmed with Sanger sequencing.

**Gene symbol and AA Change**	**Forward primer**	**Reverse primer**	**N of cases**
**STAT5B-V712E**	GGGCACTTTGTCCTTCTACA	TGTGGGTACATGTTATAGTGAGC	3
ATAX3G306delinsQQQQQQQG	TGGTGAGCAGGCCTTACCTA	ACCAGTGACTACTTTGATTCGTGA	**3**
**STAT5B-Q636P** and -**N642H**	TTCAAGTCTCCCAAGCGGTC	ATGGAGATTTCTATTGGAGCCAT	**1+5**
**CREBBP-C1294W**	TCCTAAAGCGGACAAACGCT	TCTGGTCTTGTGGTTCCGTG	**1**
**CREBBP-P875fs**	TCTGGGTTTGGGTAGCACTG	CCATTTCTGGTAGGGACAGGT	**1**
**SLC5A10-V118I**	TGGAGGATGCTTAGCCCCTA	TTGGCGATGTTTCCAGGCTAT	**1**
**CSMD3-T1809A**	TGATGGTGGAGGGGACAAAA	TGTTTGTTTTGTTGAGTAGGAGAAT	**1**
**CSMD3-V2551L**	3GAATCCTCGATCACAGGCCC	AAGCACTAGTCGCTGTATAGGTG	**1**
**DLGAP3-Q21X**	AGGGGCCCTTCAGGAGAAAT	GGTTTTAGGTGAAGAGGGCTGT	**1**
**DLGAP3-R422X**	ACTGGCACCCACTCTACACA	ACATCAAAGCCATGGGGGAT	**1**
**SHB3-H413fs** and **R415fs**	GAGTTCAGGGTCCTAGAGGGA	GAGACGACTACCACGTAGCTG	**1**
**SMCHD1-G821V**	TGACCCCCAATGGCTTTTCT	CCTTGCTTCAAGAACTGGCTG	**1**
**SMCHD1-L1575Q**	GGGTAGTGGTGGTGGTGATAA	GTAGCCGGGGCTCAAATACAA	**1**
**RYR2-A2194T**	AGACCTCAACGTATGAACTCAA	ACTTACCAAGACCAACACTGCT	**1**
**RYR2-V1488L**	AGCATGAGCTTGTTGCTTGC	TCACTGACCGCGTACCTGAT	**1**
**FNDC1-V654L**	CTCTCATCGTCCTTCCCTGC	GGCTGAGGCACTGGAATCTT	**1**
**NFK-B-G929S**	TTGTGCTTTCTCCCCTCAGAC	CGGTGTGGGAAATTGTCAGC	**1**
**AKT1-R76C**	CTCCACATGGAAGGTGCGT	TGGGTGGTATGCAAGGGGAG	**1**
**AKT1-R222S**	GGTGCCATGGAGAGTAGCC	GCAGGCCCTGAAGTACTCTT	**1**
**E2F1-R165P**	CAGCCACTGGATGTGGTTCT	CCTGTCTTGCCTGACCACAC	**1**
**JAK-1-F838V-R**	GTTTCTCCCCAAGCTGGGTT	GGTTTCTTCCATGTGCCCCT	**1**
**KMT2D-C1400Y**	GGCCAGGACAAGGAACTAGG	TGGATCTGAGTGGGATGGGG	**1**
SDK2-V293**I** and **SDK2-R268Q**	GACAAACTGAGGCGGTTCTG	AAGGACGGGGTATTGCTGTC	**1+1**
SDK2-R383H3	GGGAAGGCCCAACTTACTGG	AGACACCAAGATTCACCAGGG	**1**
SDK2-.R524fs-F	CTAATGTGGACACTCGCCCA	ATGGTATCCACCCAGAGTCCA	**1**
SHANK3-R1217C	AAGTCACCCGAGGACAAGAA	TTCACAGCAAACACCAGCTCT	**1**

Supplemetary Table 3: Amplicon design for Ion torrent targeted deep sequencing. Amplicons covering the whole coding sequence (CDS) of SETD2, CREBPP and JAK1 together with recurrent hotspot mutations in other JAK and STAT family members are indicated. The number of amplicons and the number of bases covered is also reported.

Gene Symbol	Chrom	Type	Hot Spots AA position	Start	End	Number of Amplicons	Total_Bases
SETD2	Chr 3	Whole CDS	**-**	-	-	94	8115
CREBPP	Chr 16	Whole CDS	**-**	-	-	99	7949
JAK1	Chr 1	Whole CDS	**-**	-	-	57	3945
STAT5B	Chr 17	Region	**433**	40369255	40369265	1	10
STAT5B	Chr 17	Region	**596**	40362303	40362313	1	10
STAT5B	Chr 17	Region	**636**	40359741	40359751	1	10
STAT5B	Chr 17	Region	**628**	40362207	40362217	1	10
STAT5B	Chr 17	Region	**642**	40359724	40359734	1	10
STAT5B	Chr 17	Region	**648**	40359706	40359716	1	10
STAT5B	Chr 17	Region	**659**	40359673	40359683	1	10
STAT5B	Chr 17	Region	**665**	40359654	40359664	1	10
STAT5B	Chr 17	Region	**704**	40354789	40354799	1	10
STAT5B	Chr 17	Region	**706**	40354782	40354792	1	10
STAT5B	Chr 17	Region	**712**	40354455	40354465	1	10
STAT1	Chr 2	Region	**373**	191851769	191851779	1	10
STAT1	Chr 2	Region	**389**	191851630	191851640	1	10
STAT1	Chr 2	Region	**563**	191844533	191844543	1	10
STAT3	Chr 17	Region	**614**	40475063	40475073	1	10
STAT3	Chr 17	Region	**616**	40475059	40475069	1	10
STAT3	Chr 17	Region	**618**	40475053	40475063	1	10
STAT3	Chr 17	Region	**640**	40474477	40474487	1	10
STAT3	Chr 17	Region	**647**	40474456	40474466	1	10
STAT3	Chr 17	Region	**657**	40475320	40475330	1	10
STAT3	Chr 17	Region	**658**	40474422	40474432	1	10
STAT3	Chr 17	Region	**661**	40474415	40474425	1	10
STAT5A	Chr 17	Region	**634**	40459732	40459742	1	10
JAK2	Chr 9	Region	**112**	5044412	5044422	1	10
JAK2	Chr 9	Region	**514**	5069947	5069957	1	10
JAK2	Chr 9	Region	**537**	5070017	5070032	1	15

JAK2	Chr 9	Region	**538**	5070018	5070032	1	14
JAK2	Chr 9	Region	**539**	5070020	5070032	1	12
JAK2	Chr 9	Region	**540**	5070026	5070045	1	19
JAK2	Chr 9	Region	**541**	5070028	5070043	1	19
JAK2	Chr 9	Region	**542**	5070030	5070045	1	15
JAK2	Chr 9	Region	**543**	5070030	5070045	1	15
JAK2	Chr 9	Region	**546**	5070044	5070057	1	13
JAK2	Chr 9	Region	**547**	5070047	5070058	1	11
JAK2	Chr 9	Region	**562**	5072531	50725411	1	10
JAK2	Chr 9	Region	**564**	5072536	5072546	1	10
JAK2	Chr 9	Region	**570**	5072555	5072565	1	10
JAK2	Chr 9	Region	**571**	5072556	5072566	1	10
JAK2	Chr 9	Region	**579**	5072580	5072590	1	10
JAK2	Chr 9	Region	**606**	5073734	5073744	1	10
JAK2	Chr 9	Region	**611**	5073748	5073758	1	10
JAK2	Chr 9	Region	**617**	5073765	5073775	1	10
JAK2	Chr 9	Region	**618**	5073768	5073778	1	10
JAK2	Chr 9	Region	**620**	5073776	5073786	1	10
JAK2	Chr 9	Region	**682**	5078352	5078363	1	11
JAK2	Chr 9	Region	**683**	5078355	5078367	1	12
JAK2	Chr 9	Region	**867**	5089697	5089707	1	10
JAK2	Chr 9	Region	**873**	5089714	5089724	1	10
JAK2	Chr 9	Region	**1108**	5126710	5126720	1	10
JAK3	Chr 19	Region	**172**	17953882	17953892	1	10
JAK3	Chr 19	Region	**251**	17953230	17953240	1	10
JAK3	Chr 19	Region	**271**	17953166	17953176	1	10
JAK3	Chr 19	Region	**403**	17951080	17951090	1	10
JAK3	Chr 19	Region	**501**	17949133	17949143	1	10
JAK3	Chr 19	Region	**507**	17949115	17949125	1	10
JAK3	Chr 19	Region	**511**	17949103	17949113	1	10
JAK3	Chr 19	Region	**563**	17948741	17948751	1	10
JAK3	Chr 19	Region	**572**	17948004	17948014	1	10
JAK3	Chr 19	Region	**573**	17948001	17948011	1	10

JAK3	Chr 19	Region	**657**	17945964	17945974	1	10
JAK3	Chr 19	Region	**674**	17945913	17945923	1	10
JAK3	Chr 19	Region	**722**	17945691	17945701	1	10
JAK3	Chr 19	Region	**857**	17943433	17943443	1	10

Supplementary Table 4: Somatic non-silent mutations validated by targeted resequencing in the 15 samples analyzed by WES. Some regions could not be effectively targeted and were not confirmed (not covered).

Gene	SETD2		CREBBP		STAT5B		JAK3		JAK1		STAT3		JAK2		STAT1		STAT5A	
Plattaform	**WES**	**Ion Torrent**	WES	Ion Torrent	WES	Ion Torrent	WES	Ion Torrent	WES	Ion Torrent	WES	Ion Torrent	WES	Ion Torrent	WES	Ion Torrent	WES	Ion Torrent
EATL-II-01	WT	WT	WT	WT	WT	WT	WT	WT	WT	WT	WT	WT	WT	WT	WT	WT	WT	WT
EATL-II-02	Splicing exon 20-21<br>p.C1533Y	Not covered<br>p.C1533Y	WT	WT	WT	WT	WT	WT	WT	WT	WT	WT	WT	WT	WT	WT	WT	WT
EATL-II-03	p.T2037fs	p.T2037fs	p.C1294W	p.C1294W	WT	WT	WT	WT	WT	WT	WT	WT	WT	WT	WT	WT	WT	WT
EATL-II-04	Splicing exon 13-14	Not covered	WT	WT	WT	WT	p.A573V	p.A573V	WT	WT	WT	WT	WT	WT	WT	WT	WT	WT
EATL-II-12	p.N1643K	p.N1643K	WT	WT	p.V712E	p.V712E	WT	WT	WT	WT	WT	WT	WT	WT	WT	WT	WT	WT
EATL-II-13	WT	WT	WT	WT	p.N642H	p.N642H	p.A573V	p.A573V	p.N5S	p.N5S	WT	WT	WT	WT	WT	WT	WT	WT
EATL-II-14	p.M1607I<br>p.R1459X	p.M1607I<br>p.R1459X	WT	WT	WT	WT	p.M511I<br>p.P676R	p.M511I<br>p.P676R	WT	WT	WT	WT	WT	WT	WT	WT	WT	WT
EATL-II-15	p.A1597fs	p.A1597fs	p.P875fs	p.P875fs	p.N642H	p.N642H	WT	WT	WT	WT	WT	WT	WT	WT	WT	WT	WT	WT
EATL-II-17	p.P1934fs<br>p.K2546fs	p.P1934fs<br>p.K2546fs	Splicing exone 10-11	Splicing exone 10-11	p.N642H	p.N642H	p.R657W	p.R657W	WT	WT	WT	WT	WT	WT	WT	WT	WT	WT
EATL-II-20	p.F573fs<br>Splicing exon1-2	p.F573fs<br>Not covered	WT	WT	p.N642H	p.N642H	p.A573V	p.A573V	WT	WT	WT	WT	WT	WT	WT	WT	WT	WT
EATL-II-21	p.L2486R	p.L2486R	WT	WT	WT	WT	p.563_566del	p.563_566del	WT	WT	WT	WT	WT	WT	WT	WT	WT	WT
EATL-II-22	p.R2121delinsX<br>p.Y1579N	p.R2121delinsX<br>p.Y1579N	WT	WT	p.V712E	p.V712E	p.M511I	p.M511I	WT	WT	WT	WT	WT	WT	WT	WT	WT	WT
EATL-II-24	p.G975X	p.G975X	WT	WT	p.N642H<br>p.Q636P	p.N642H<br>p.Q636P	WT	WT	WT	WT	WT	WT	WT	WT	WT	WT	WT	WT
EATL-II-25	p.C530fs<br>p.Y2543X	p.C530fs<br>p.Y2543X	p.Q2355fs	p.Q2355fs	p.V712E	p.V712E	WT	WT	p.F838V	p.F838V	WT	WT	WT	WT	WT	WT	WT	WT
EATL-II-26	p.S1624C<br>p.T2388fs	p.S1624C<br>p.T2388fs	WT	WT	p.T628S<br>p.Q706L	p.T628S<br>p.Q706L	WT	WT	WT	WT	WT	WT	WT	WT	WT	WT	WT	WT

Supplementary Table 5: Somatic non-silent mutations identified by targeted sequencing for all the genes included in the Ion torrent gene panel in 8 EATL-II samples and 8 EATL-I samples not examined by WES

Patient ID	Chrom	Start	End	REF	ALT	Gene Symbol	Variant allele frequency	Mutation type	Transcript ID:Exon:Codon changes:AA changes	dbSNP Build 138	Allele freq. 1000Genome project*	Allele freq. Exome Sequencing Project §	COSMIC ID v.70	SIFT score	SIFT Prediction	PolyPhen-2 HDIV† score	PolyPhen-2 HDIV† Prediction
EATL-II-5	3	47088067	47088067	-	AT	*SETD2*	22.99%	frameshift insertion	SETD2:NM_014159:exon16:c.7007_7008insAT:p.I2336fs	0	0	0	0	0	Damaging	0	0
EATL-II-6	1	65312365	65312365	A	G	*JAK1*	17.47%	nonsynonymous SNV	JAK1:NM_002227:exon14:c.T1954C:p.Y652H	0	0	0	ID=COSM96408;OCCURENCE=1(haematopoietic_and_lymphoid_tissue)	0.5	Tolerated	0.924	Probably damaging
EATL-II-6	19	17949108	17949108	C	T	*JAK3*	56.24%	nonsynonymous SNV	JAK3:NM_000215:exon11:c.G1533A:p.M511I	0	0	0	ID=COSM1318360,COSM51374;OCCURENCE=1(large_intestine),9(haematopoietic_and_lymphoid_tissue)	0.12	Tolerated	0.187	Benign
EATL-II-7	3	47165268	47165268	A	-	*SETD2*	39.12%	frameshift deletion	SETD2:NM_014159:exon3:c.858delT:p.I286fs	0	0	0	0	0	0	0	0
EATL-II-7	17	40359729	40359729	T	G	*STAT5B*	49.34%	nonsynonymous SNV	STAT5B:NM_012448:exon16:c.A1924C:p.N642H	0	0	0	ID=COSM1716590;OCCURENCE=2(haematopoietic_and_lymphoid_tissue)	0.19	Tolerated	0.999	Damaging
EATL-II-9	3	47147507	47147507	T	C	*SETD2*	76.55%	nonsynonymous SNV	SETD2:NM_014159:exon6:c.A4819G:p.M1607V	0	0	0	0	0.03	Damaging	0.997	Damaging
EATL-II-9	17	40359729	40359729	T	G	*STAT5B*	34.23%	nonsynonymous SNV	STAT5B:NM_012448:exon16:c.A1924C:p.N642H	0	0	0	ID=COSM1716590;OCCURENCE=2(haematopoietic_and_lymphoid_tissue)	0.19	Tolerated	0.999	Damaging
EATL-II-10	3	47162927	47162927	G	A	*SETD2*	18.18%	stopgain	SETD2:NM_014159:exon3:c.C3199T:p.Q1067X	0	0	0	ID=COSM1692710,COSM1692709;OCCURENCE=1(skin)	0	Damaging	0	0
EATL-II-10	17	40359729	40359729	T	G	*STAT5B*	31.78%	nonsynonymous SNV	STAT5B:NM_012448:exon16:c.A1924C:p.N642H	0	0	0	ID=COSM1716590;OCCURENCE=2(haematopoietic_and_lymphoid_tissue)	0.19	Tolerated	0.999	Damaging
EATL-II-19	3	47059132	47059132	C	G	*SETD2*	64.41%	nonsynonymous SNV	SETD2:NM_014159:exon20:c.G7529C:p.R2510P	0	0	0	ID=COSM3365340,COSM3365339;OCCURENCE=1(kidney)	0	Damaging	0.999	Damaging
EATL-II-19	17	40359729	40359729	T	G	*STAT5B*	37.08%	nonsynonymous SNV	STAT5B:NM_012448:exon16:c.A1924C:p.N642H	0	0	0	ID=COSM1716590;OCCURENCE=2(haematopoietic_and_lymphoid_tissue)	0.19	Tolerated	0.999	Damaging
EATL-II-28	17	40359729	40359729	T	G	*STAT5B*	52.52%	nonsynonymous SNV	STAT5B:NM_012448:exon16:c.A1924C:p.N642H	0	0	0	ID=COSM1716590;OCCURENCE=2(haematopoietic_and_lymphoid_tissue)	0.19	Tolerated	0.999	Damaging
EATL-II-28	19	17948006	17948006	G	A	*JAK3*	51.03%	nonsynonymous SNV	JAK3:NM_000215:exon13:c.C1718T:p.A573V	0	0	0	ID=COSM34215;OCCURENCE=6(haematopoietic_and_lymphoid_tissue)	0	Damaging	0.999	Damaging
EATL-II-30	3	47129722	47129722	C	A	*SETD2*	39.19%	stopgain	SETD2:NM_014159:exon10:c.G5158T:p.E1720X	0	0	0	ID=COSM480150,COSM480149;OCCURENCE=1(kidney)	0.18	Tolerated	0	0
EATL-II-30	17	40354787	40354787	T	A	*STAT5B*	61.9%	nonsynonymous SNV	STAT5B:NM_012448:exon17:c.A2117T:p.Q706L	0	0	0	0	0.1	Tolerated	0.656	Probably damaging

EATL-II-30	19	17945918	17945918	A	G	*JAK3*	25.68%	nonsynonymous SNV	JAK3:NM_000215:exon15:c.T2021C:p.V674A	0	0	0	ID=COSM327317;OCCURENCE=2(haematopoietic_and_lymphoid_tissue)	0	Damaging	0.996	Damaging
EATL-I-31	1	65301158	65301158	C	A	*JAK1*	70.87%	nonsynonymous SNV	JAK1:NM_002227:exon24:c.G3290T:p.G1097V	0	0	0	0	NA	Damaging	NA	Damaging
EATL-I-32	1	65301159	65301159	C	A	*JAK1*	32.69%	nonsynonymous SNV	JAK1:NM_002227:exon24:c.G3289T:p.G1097C	0	0	0	0	0.01	Damaging	NA	Damaging
EATL-I-33	1	65301158	65301158	C	T	*JAK1*	4.36%	nonsynonymous SNV	JAK1:NM_002227:exon24:c.G3290A:p.G1097D	0	0	0	0	0.22	Tolerated	1	Damaging
EATL-I-38	1	65301159	65301159	C	T	*JAK1*	45.53%	nonsynonymous SNV	JAK1:NM_002227:exon24:c.G3289A:p.G1097S	0	0	0	0	0.21	Tolerated	0.999	Damaging
EATL-I-38	17	40474427	40474427	C	A	*STAT3*	46.04%	nonsynonymous SNV	STAT3:NM_139276:exon21:c.G1974T:p.K658N	0	0	0	ID=COSM1155742;OCCURENCE=2(haematopoietic_and_lymphoid_tissue)	0.07	Tolerated	0.99	Damaging
EATL-I-39	17	40474461	40474461	T	A	*STAT3*	24.14%	nonsynonymous SNV	STAT3:NM_139276:exon21:c.A1940T:p.N647I	0	0	0	ID=COSM1155744;OCCURENCE=12(haematopoietic_and_lymphoid_tissue)	NA	NA	NA	Benign

* Allele frequency observed in the 1000 Genome project (Oct 2014)

§ Allele frequency observed in the European American subjects in the NHLBI GO Exome Sequencing Project - ESP Dic 2014)

† HDIV: HumDiv PolyPhen-2 prediction model

Supplementary Table 6: Summary of SETD2 analysis in the extended cohort of intestinal lymphomas (15+14 EATL-II, 16 EATL-I and 8 intestinal non-Hodgkin lymphomas of various histotypes). FISH results are expressed as the percentage of nuclei with SETD2 (3p21.31) locus deletion and as the ratio of red to green signals, corresponding to 3p21.31 (RP11-425J9) and 3p25 (RP11-266J6/RP11-485N3) probes, respectively, except for case 25 where 3p21.31 loss was evidenced with RP11-650F17 probe.
DLBCL: diffuse large B-cell lymphoma; FL: follicular lymphoma; PTLD: post-transplantation lymphoproliferative disorder; ALK: anaplastic lymphoma kinase; ALCL: anaplastic large cell lymphoma; FL: follicular lymphoma; PTCL-NOS: peripheral T-cell lymphoma, not otherwise specified. NA: not analyzed; NC: not contributive. In red mutations in SETD2 discovered by targeted resequencing.

**Diagnosis**	**Case**	**Whole exome sequencing**	**Targeted Deep Sequencing**	**SETD2 mutation**	***SETD2* locus CNV (3p21.31)**	**% nuclei with *SETD2* locus deletion (cut-off: 11.2%)**	**FISH ratio 3p21.31/3p25**	**SETD2 IHC score**	**Total H3 IHC score**	**H3K36me2 IHC score**	**H3K36me3 IHC score**	**H3K36me3/me2 ratio**
**EATL-II**	CASE 1	yes	yes	WT	heterozygous deletion	81.3	0.56	4	12	8	8	1
**EATL-II**	CASE 2	yes	yes	splicing+p.C1533Y	no deletion	5.5	1.01	6	12	8	2	0.25
**EATL-II**	CASE 3	yes	yes	p.T2037fs	no deletion	1.9	1	1	12	6	1	0.17
**EATL-II**	CASE 4	yes	yes	splicing	heterozygous deletion	82.9	0.51	0	NA	8	1.5	0.19
**EATL-II**	CASE 5	no	no	p.I2336fs	no deletion	2.9	1.01	0.25	NA	10	1.5	0.15
**EATL-II**	CASE 6	no	no	WT	no deletion	2.0	1.01	2	NA	4.5	4.5	1
**EATL-II**	CASE 7	no	no	p.I286fs	no deletion	4.0	1.03	0	NA	6	1	0.17
**EATL-II**	CASE 8	no	no	NA	no deletion	4.0	1.02	NC	NA	12	3	0.25
**EATL-II**	CASE 9	no	no	p.M1607V	heterozygous deletion	72.9	0.58	1	NA	3	0	0
**EATL-II**	CASE 10	no	no	p.Q1067X	NC	NC	NC	0.5	NA	7.5	0	0
**EATL-II**	CASE 11	no	no	NA	NC	NC	NC	NC	NA	6	0	0
**EATL-II**	CASE 12	yes	yes	p.N1643K	heterozygous deletion	68.2	0.61	0.5	12	12	6	0.5
**EATL-II**	CASE 13	no	no	WT	no deletion	0.0	1.02	10	12	10	12	1.2
**EATL-II**	CASE 14	yes	yes	p.M1607I+p.R1459X	no deletion	5.2	1.01	0	12	10	1	0.1
**EATL-II**	CASE 15	yes	yes	p.A1597fs	no deletion	6.4	1	NC	12	4.5	0	0
**EATL-II**	CASE 16	no	no	NA	NC	NC	NC	NC	NA	10	1	0.1
**EATL-II**	CASE 17	yes	yes	p.K2546fs+p.P1934fs	no deletion	1.0	1.03	0	NA	6	0	0
**EATL-II**	CASE 18	no	no	NA	NC	NC	NC	NC	NA	NC	NC	NC
**EATL-II**	CASE 19	no	no	p.R2510P	no deletion	3.2	1	8	NA	10	0	0
**EATL-II**	CASE 20	yes	yes	p.F573fs+splicing	no deletion	7.9	1	1	12	12	1	0.08
**EATL-II**	CASE 21	yes	yes	p.L2486R	no deletion	7.5	1.02	2	NA	8	0.5	0.06
**EATL-II**	CASE 22	yes	yes	p.R2121X+p.Y1579N	no deletion	5.5	1	2	12	8	12	1.5
**EATL-II**	CASE 23	no	no	NA	no deletion	1.1	0.98	NC	NA	8	0	0
**EATL-II**	CASE 24	yes	no	p.G975X	heterozygous deletion	64.0	0.63	NC	12	6	0	0
**EATL-II**	CASE 25	yes	no	p.Y2543X+p.C530fs	no deletion	3.9	1	0.5	NA	4.5	0	0
**EATL-II**	CASE 26	yes	no	p.T2388fs+p.S1624C	no deletion	1.9	1	NC	NA	NC	NC	NC
**EATL-II**	CASE 27	no	no	NA	NC	NC	NC	0	NA	8	1	0.125

**EATL-II**	CASE 28	no	yes	WT	heterozygous deletion	68.5	0.55	8	12	6	0	0
**EATL-II**	CASE 30	no	yes	p.E1720X	heterozygous deletion	62.1	0.63	1	12	8	0	0
**EATL-I**	CASE 31	no	yes	WT	no deletion	2.0	1.01	12	12	12	12	1
**EATL-I**	CASE 32	no	yes	WT	no deletion	2.0	0.99	10	12	6	10	1.67
**EATL-I**	CASE 33	no	yes	WT	no deletion	2.8	1	10	12	10	10	1
**EATL-I**	CASE 34	no	yes	WT	NC	NC	NC	8	12	10	10	1
**EATL-I**	CASE 41	no	no	NA	no deletion	4.9	1.03	8	12	8	10	1.25
**EATL-I**	CASE 42	no	no	NA	NC	NC	NC	6	12	10	10	1
**EATL-I**	CASE 43	no	no	NA	NC	NC	NC	4.5	12	6	4	0.67
**EATL-I**	CASE 37	no	yes	WT	NC	NC	NC	NC	NA	10	6	0.6
**EATL-I**	CASE 44	no	no	NA	no deletion	3.9	1.02	8	12	12	8	0.67
**EATL-I**	CASE 45	no	no	NA	no deletion	0.0	1.01	8	NA	10	10	1
**EATL-I**	CASE 38	no	yes	WT	no deletion	8.9	0.94	12	NA	10	8	0.8
**EATL-I**	CASE 46	no	no	NA	no deletion	5.9	1	10	NA	12	12	1
**EATL-I**	CASE 47	no	no	NA	no deletion	0.0	1.1	8	12	10	10	1
**EATL-I**	CASE 48	no	no	NA	no deletion	2.0	1.02	10	12	10	8	0.8
**EATL-I**	CASE 39	no	yes	WT	NC	NC	NC	12	NA	12	12	1
**EATL-I**	CASE 40	no	yes	WT	no deletion	1.0	1.05	10	12	12	12	1
**DLBCL**	CASE 35	no	yes	WT	no deletion	0.0	1.05	10	12	2	6	3
**Burkitt**	CASE 49	no	no	NA	no deletion	4.6	1.08	12	12	10	12	1.2
**DLBCL**	CASE 50	no	no	NA	no deletion	2.9	1.01	12	12	12	12	1
**DLBCL/PTLD**	CASE 51	no	no	NA	no deletion	0.0	2.66	12	12	10	12	1.2
**FL**	CASE 52	no	no	NA	no deletion	1.0	1.06	8	12	7.5	12	1.6
**PTCL NOS CD30+**	CASE 53	no	no	NA	no deletion	0.0	1.33	10	NA	10	10	1
**ALK+ ALCL**	CASE 36	yes	yes	WT	no deletion	1.0	1.06	10	12	10	12	1.2
**ENKTCL**	CASE 54	no	no	NA	no deletion	0.0	1.09	12	12	12	12	1
